# Testing the Pragmatic Effectiveness of a Consumer-Based Mindfulness Mobile App in the Workplace: Randomized Controlled Trial

**DOI:** 10.2196/38903

**Published:** 2022-09-28

**Authors:** Jennifer L Huberty, Hallie M Espel-Huynh, Taylor L Neher, Megan E Puzia

**Affiliations:** 1 Calm.com, Inc. San Francisco, CA United States; 2 College of Health Solutions Arizona State University Phoenix, AZ United States; 3 Center for the Study of Aging Boise State University Boise, ID United States; 4 Behavioral Research and Analytics, LLC Salt Lake City, UT United States

**Keywords:** mindfulness, mobile apps, workforce, workplace, presenteeism, mental health

## Abstract

**Background:**

Mental health and sleep problems are prevalent in the workforce, corresponding to costly impairment in productivity and increased health care use. Digital mindfulness interventions are efficacious in improving sleep and mental health in the workplace; however, evidence supporting their pragmatic utility, potential for improving productivity, and ability to reduce employer costs is limited.

**Objective:**

This pragmatic, cluster randomized controlled trial aimed to evaluate the experimental effects of implementing a commercially available mindfulness app—Calm—in employees of a large, multisite employer in the United States. Outcomes included mental health (depression, anxiety, and stress), sleep (insomnia and daytime sleepiness), resilience, productivity impairment (absenteeism, presenteeism, overall work impairment, and non–work activity impairment), and health care use (medical visit frequency).

**Methods:**

Employees were randomized at the work site to receive either the Calm app intervention or waitlist control. Participants in the Calm intervention group were instructed to use the Calm app for 10 minutes per day for 8 weeks; individuals with elevated baseline insomnia symptoms could opt-in to 6 weeks of sleep coaching. All outcomes were assessed every 2 weeks, with the exception of medical visits (weeks 4 and 8 only). Effects of the Calm intervention on outcomes were evaluated via mixed effects modeling, controlling for relevant baseline characteristics, with fixed effects of the intervention on outcomes assessed at weeks 2, 4, 6, and 8. Models were analyzed via complete-case and intent-to-treat analyses.

**Results:**

A total of 1029 employees enrolled (n=585 in the Calm intervention group, including 101 who opted-in to sleep coaching, and n=444 in waitlist control). Of them, 192 (n=88 for the Calm intervention group and n=104 for waitlist) completed all 5 assessments. In the complete-case analysis at week 8, employees at sites randomized to the Calm intervention group experienced significant improvements in depression (*P*=.02), anxiety (*P*=.01), stress (*P*<.001), insomnia (*P*<.001), sleepiness (*P*<.001), resilience (*P*=.02), presenteeism (*P*=.01), overall work impairment (*P*=.004), and nonwork impairment (*P*<.001), and reduced medical care visit frequency (*P*<.001) and productivity impairment costs (*P*=.01), relative to the waitlist control. In the intent-to-treat analysis at week 8, significant benefits of the intervention were observed for depression (*P*=.046), anxiety (*P*=.01), insomnia (*P*<.001), sleepiness (*P*<.001), nonwork impairment (*P*=.04), and medical visit frequency (*P*<.001).

**Conclusions:**

The results suggest that the Calm app is an effective workplace intervention for improving mental health, sleep, resilience, and productivity and for reducing medical visits and costs owing to work impairment. Future studies should identify optimal implementation strategies that maximize employee uptake and large-scale implementation success across diverse, geographically dispersed employers.

**Trial Registration:**

ClinicalTrials.gov NCT05120310; https://clinicaltrials.gov/ct2/show/NCT05120310

## Introduction

### Mental Health Concerns in the Workplace

Approximately 8% to 15% of the US adult population is affected by depression and anxiety, respectively [1,2], at least 1 in 4 is affected negatively by stress, and ≥60% report sleep disturbance such as trouble falling or staying asleep, insufficient sleep duration, or restlessness during the night [3]. Poor mental health (ie, depression, anxiety, or stress) and lack of sleep are linked to limitations in daily activities and increased health care use and thus pose a major burden to society [4-13]. Poor mental health and sleep disturbance are increasingly recognized as top contributors to reduced productivity and increased economic burden for employers (ie, via increased medical costs per employee and financial losses due to reduced work productivity) [14-16]. In the United States, depressive symptoms alone account for an estimated US $6 billion total in missed work costs (absenteeism) and nearly US $85 billion in productivity losses due to on-the-job impairment (presenteeism) [16,17]. Sleep-related productivity losses are estimated to cost more than US $1967 *per employee* each year [16].

### Benefits of Mindfulness and Gaps in the Literature

Mindfulness-based workplace interventions (eg, mindfulness meditation or mindfulness-based stress reduction programs) have been shown to improve mental health (ie, depression, anxiety, and stress) and sleep [18-20]. Two recent meta-analyses examined the effects of mindfulness-based interventions in the workplace on a number of employee mental health outcomes and sleep. Mindfulness interventions have demonstrated significant benefits in anxiety, stress, and sleep [18,20]. One of the meta-analyses reported marginal improvements in depressive symptoms as a result of mindfulness interventions but noted limitations such as the small number of studies available and data inadequacy [20]. The other reported on another key construct in emotional coping–resilience–which refers to one’s ability to *bounce back* or recover from stress [21,22] and which can serve as a buffer between stressful life events and the development of mental health problems including depression, anxiety, and chronic stress [23]. Meta-analytic results indicated significant effects of mindfulness interventions on resilience in the workplace, although the results were interpreted with caution because only 4 studies contributed to the analysis [22].

Even fewer studies have examined the effects on key work productivity measures, such as absenteeism (productivity losses owing to missed time working as a result of mental or physical health concerns), presenteeism (productivity losses due to impairment while on the job as a result of mental or physical health concerns), overall impairment in work productivity (absenteeism and presenteeism combined), and overall impairment in nonwork activities [18,22,24]; all of these constructs are particularly relevant to balancing the costs and benefits of these programs for employers. Some studies have found positive effects of mindfulness training on productivity [25,26], particularly for presenteeism, whereas others have found no significant impact [27,28]. One potential reason for these mixed results is the heterogeneity of measurement and the use of assessment measures that have not been extensively validated [22]. There is also a dearth of evidence for the ultimate cost-effectiveness of these programs in terms of health care expenditures for employers [18,22]. Thus, more work is needed in this area to understand the economic benefits of mindfulness-based interventions in the workplace, particularly with well-validated measures of overall productivity.

In addition, to date, most mindfulness-based interventions in the workplace have been delivered in-person, which limits scalability because of the costly need for a trained interventionist at each session [29], as well as employee-level barriers such as the need for child care, transportation to a site, and limited scheduling options [30,31]. Digital mindfulness-based interventions in the workplace offer employers the ability to help employees manage their mental health, while mitigating many barriers associated with in-person interventions. Although few studies have evaluated mindfulness apps specifically in the workplace, preliminary evidence supports their effectiveness both for mental health and productivity outcomes. For example, 1 recent study found that employees randomized to receive a commercially available mindfulness meditation app in the workplace had significantly greater improvements in anxiety and depression versus those in a waitlist control condition, but no effects on sleep were evaluated [32]. The study also found positive effects on self-reported job strain but did not assess employee productivity [32]. Another limitation is that the study sample was restricted to a relatively healthy population due to the exclusion of employees with several common physical and mental health concerns (eg, depression, hypertension, and cardiovascular disease) [32]. It is unclear how the results would generalize to the broader population of working individuals with a range of health concerns. Another recent study examined the effects of a mindfulness app in the workplace compared with an app-plus-group intervention or waitlist control; however, this study did not evaluate mental health or sleep outcomes aside from post hoc participant perceptions [28]. In addition, there was only a marginal effect on measured overall productivity, and the study did not examine presenteeism and absenteeism separately [28]. To date, no studies have assessed the effects of a stand-alone mindfulness app concurrently on mental health outcomes, resilience, workplace productivity, and health care use, particularly with an inclusive sample that is more representative of the present-day workforce.

### Study Aims

The purpose of this study was to evaluate the experimental effects of the Calm app, a mobile meditation app, on employee mental health (depression, anxiety, and stress), sleep, resilience, work productivity outcomes (absenteeism, presenteeism, overall work impairment, and overall activity impairment) and health care use (operationalized as the number of visits with a medical provider) in a workplace setting. We also explored the financial benefit of the Calm app in terms of productivity impairment cost savings (the amount of money saved per employee owing to the improvements in overall work productivity observed in the Calm intervention group vs waitlist control). This is the first study to evaluate the Calm app in a workplace setting (offered to employees for free and paid by the employer), specifically with a focus on employee mental health and productivity.

## Methods

### Ethics Approval

This study was approved by the institutional review board of Arizona State University (STUDY00014072) and registered with ClinicalTrials.gov (trial registration NCT05120310). All participants provided electronic informed consent before participating in the study.

### Participants and Recruitment

Participants were employees of a large consumer electronics retailer. Recruitment occurred nationally between August and December 2021 via email invitations from human resources, store leaders, and flyers posted in store breakrooms. Email materials and flyers included a QR code and website link that directed participants to a web-based eligibility survey (via the Qualtrics web-based survey platform). All recruitment materials referred to the intervention as the use of a *health and wellness app*. Invitations and flyers for recruitment were sent to 294 (estimated 20,000 employees), 288 (estimated 18,000 employees), and 511 work sites (estimated 36,000 employees) over the 6-month recruitment period.

Employees were eligible for the study if they were (1) were a current employee of the company, (2) were at least 18 years of age, (3) were able to read and understand English, (4) owned a smartphone, (5) were willing to download the Calm app, and (6) were meditation-naive and had not practiced meditation for ≥60 minutes per month for the past 6 months. We included only those that were meditation-naive because the literature suggests differences in the effects of mindfulness in those that have less meditation experience than those that have more (eg, recruiting different brain regions during meditation, which are differentially related to building attentional control and emotional regulation, versus maintaining existing networks [33]). Eligibility surveys took approximately 2 minutes to complete. At the end of the survey, ineligible employees were notified of their status, and eligible employees were automatically directed to a link containing the electronic informed consent and a video explaining the details of the study and the consent form. After consenting, participants were directed to complete the baseline questionnaires.

Depending on site randomization, employees were assigned to either the intervention group (ie, Calm app [10 minutes per day]) or the waitlist control group (ie, received access to the Calm app after 8 weeks). The primary and secondary outcomes of this study were sleep (ie, insomnia symptoms, daytime sleepiness, and sleep diaries), productivity (ie, absenteeism, presenteeism, work impairment, and activity impairment), resilience, and mental health (ie, depression, anxiety, and stress). Study outcomes were assessed at baseline (week 0), midintervention (weeks 2, 4, and 6), and after the intervention (week 8). A subsample of participants who self-reported elevated sleep disturbance was also invited to receive 6 weeks of sleep coaching during their study participation. Coaching participants completed daily sleep diaries to measure sleep or wake time and sleep quality (secondary outcomes). The sleep coaching outcomes are beyond the scope of this manuscript and will be reported elsewhere.

### Randomization and Blinding

Randomization occurred at the work site to avoid treatment contamination between employees at the same work site. Before recruitment, all sites in the company (N=1096 sites) were randomized using stratification by total number of employees (ie, 33rd and 67th percentiles; small ≤53 employees, medium=54-73 employees, and large ≥74 employees). Store locations were randomized using allocation sequences generated before the start of the study [34]. Allocation sequences were concealed from the research personnel involved in allocation until the time of group assignment. Participants were informed of their group assignment following completion of the baseline questionnaires.

### Intervention and Control Groups

#### Calm Intervention Group

Participants assigned to the intervention group were instructed to download the consumer-based mobile meditation app, Calm, and were asked to use it autonomously for at least 10 minutes per day during the 8-week intervention period. Meditation in the Calm app uses mindfulness components [35], breathing techniques, and body scans, all of which are consistent with core mindfulness practices, including mindfulness-based stress reduction (nonjudgmental moment-to-moment awareness [36]), and vipassana (objective observation of physical sensation in the body [37]). The frequency, dose, and timing of engagement with the Calm app, as well as its content and use of features, is entirely self-selected by the user. The Calm app is offered internationally in 7 different languages. The Calm app may be accessed by purchasing the app via a subscription-based service or offered by an employer as a benefit; some content is freely available upon download. In addition to using the Calm app, participants had the option to schedule one synchronous 20-minute web-based coaching session with a Calm app coach in the first week to orient them to the Calm app. Participants received weekly SMS text message reminders to use the Calm app on Sundays at noon during the intervention. For the final survey, participants were able enter a raffle to win 1 of 5 boxes of Calm *swag* (pencils, notepads, book, etc), which were provided by the Calm app.

A sample of participants with elevated baseline scores on the Insomnia Severity Index (ISI; see the Measures section; determined as a score of >10 at baseline assessment) were invited to attend 5 weekly sessions over 6 weeks with a certified sleep coach to help them improve their sleep. Sessions were structured around basic sleep hygiene principles (eg, establishing a regular pattern of sleep, engaging in sleep hygiene practices, sleep restriction (as appropriate for insomnia symptoms), practicing bedtime mindfulness, and improving the sleep environment). The intent was to randomize individuals with elevated ISI scores to either sleep coaching or Calm app only. However, due to low enrollment, we quickly transitioned to offering sleep coaching to all participants with an elevated ISI. Those who opted into sleep coaching were asked to complete a 2-week sleep diary at the beginning and end of the 6-week period.

#### Waitlist Control Group

Participants randomized to the waitlist control group were instructed via email to continue with their usual routines during the 8-week assessment period. After week 8, they received access to the Calm app for an additional 8 weeks. Waitlisted participants with elevated baseline ISI scores were invited to participate in sleep coaching after completing their waitlist period. To ensure consistency in measurement, they also completed a sleep diary during the waitlist period.

### Measures

All participants in both groups were asked to complete electronic self-report assessments of outcomes every 2 weeks from baseline until the completion of the 8-week study period. The constructs measured and the psychometric properties of the assessments used are described.

#### Demographics

Demographics and individual characteristics (16 items assessing personal characteristics, such as race, ethnicity, work, and medical status) were collected at baseline.

#### Mental Health

Mental health was measured using the Depression Anxiety Stress Scale (DASS; DASS-21), a 21-item scale assessing symptoms of depression, anxiety, and stress over the past week [38]. The DASS-21 is the short form of the original 42-item measure by Lovibond and Lovibond [39]. It has demonstrated construct validity and maintains the tripartite factor structure of the original DASS-42, effectively distinguishing among the latent constructs of depression, anxiety, and stress via items measuring low positive affect, physiological hyperarousal, and perceived stress [38]. In general population samples, the DASS-21 has shown adequate internal consistency (Cronbach α of .88, .82, and .90 for the depression, anxiety, and stress subscales, respectively) and good convergent and discriminant validity compared with other measures of depression and anxiety [38,39].

#### Sleep

Insomnia symptoms were assessed among all participants via the ISI, a 7-item self-report questionnaire assessing insomnia symptoms (eg, difficulty falling and staying asleep) during the past 2 weeks and the distress and impairment associated with the symptoms [40]. Items are rated on a 5-point Likert-type scale; total scale scores are obtained by summing the item ratings. The ISI has demonstrated good internal consistency (Cronbach α=.74 in the validation sample), sensitivity to change, and convergence with both objectively measured sleep disturbance and clinician ratings [40]. Daytime sleepiness symptoms were measured using the Epworth Sleepiness Scale, which includes 8 items assessing recent dozing behavior during routine daytime activities (sitting and reading, in conversation, etc) [41]. Items are rated on a 4-point Likert scale from 0 (*would never doz*e) to 3 (*high chance of dozing*). The total scores were obtained by summing the item ratings (range 0-24, with higher scores indicating greater sleepiness). The Epworth Sleepiness Scale has shown high internal consistency (Cronbach α=.7 to .9 in varying populations), demonstrates convergent validity with objective measures of sleepiness and sleep disturbance (ie, sleep latency), and differentiates between clinical and nonclinical sleep populations [41,42].

#### Resilience

Resilience was measured using the Brief Resilience Scale (BRS), which measures an individual’s ability to bounce back and recover from stress [21]. Respondents are asked to rate the extent to which 6 statements related to resilience apply to them, on a 5-point Likert-type scale (ie, from *strongly disagree* to *strongly agree*). The BRS has been validated in college students as well as clinical samples (eg, individuals with chronic medical concerns) with good to excellent internal consistency (Cronbach α ranging from .80 to .91 for the overall scale) [43].

#### Work Productivity and Impairment

The Work Productivity and Activity Impairment (WPAI) Questionnaire–General Health measure (WPAI general health) is a 6-item scale that measures general physical and mental health–related impairments in work and nonwork activities as well as absenteeism and presenteeism [24]. Respondents were asked about current employment, hours missed due to health problems and other reasons, hours worked, and the degree to which health affected productivity during work and in other nonwork activities in the past 7 days. The 4 outcomes generated from the scale are percent work time missed due to health (absenteeism), percent impairment while working due to health (presenteeism), percent overall work impairment due to health (productivity impairment), and percent nonwork activity impairment due to health (nonwork activity impairment).

#### Medical Care Visits

At weeks 4 and 8, participants were asked to self-report the number of times they had seen a medical provider in the past 4 weeks.

#### Productivity Cost Savings

The average amount of money saved per employee (US $) due to the improvements in overall work productivity observed in the Calm intervention group versus waitlist control was computed using the human capital approach (HCA) [44,45], which is one of the most widely used methods for estimating the monetary value associated with productivity losses due to a specific cause (eg, mental and physical health problems) [44]. This approach assumes that 1 hour of differential productivity (ie, productivity gained via the Calm app) is equivalent in value to an individual’s wages for that same time. In this case, because the WPAI assesses work productivity and impairment over the past 7 days, the HCA produces a weekly estimate of productivity costs associated with employee impairment. Work impairment and associated productivity costs for the present analysis were derived according to employee wage type—hourly versus salaried. First, the total monetary value of an individual’s *potential* productive hours was computed. For hourly workers, the weekly wage was calculated as an employee’s hourly wage multiplied by the number of hours per week they worked, and for salaried employees, weekly wages were calculated as an employee’s annual salary divided by 52 weeks in a year (given that weekly wages among salaried employees do not depend on the number of hours worked each week). Consistent with HCA, employees’ weekly overall work impairment percentages, as indicated by the WPAI overall work impairment metric (ie, percentage of time, relative to the hours worked per week, that an employee is absent or reporting impaired productivity due to mental or physical health problems), were multiplied by their weekly wages to obtain an overall cost of absenteeism and impaired productivity for each individual. These weekly productivity cost values associated with work impairment were then included as the outcome variable in analyses via mixed effects modeling (see the Data Analyses section).

#### App Use

App use data were provided by the Calm app. Use over time was measured as the average number of sessions and minutes per week of use per employee. Use assessments included measures of overall app use (any component) and the use of specific app components (eg, meditation, music, and sleep stories).

### Data Analyses

Power analyses were conducted using G*Power 3.0. Consistent with prior research that has tested similar interventions and measured changes in mental health, resilience, and sleep [46-49], we assumed small to moderate effect sizes for improvements (conservative estimate of Cohen *d*=0.12), thus estimating a total needed sample size of 364. Anticipating approximately 30% attrition, we aimed to enroll a minimum of 500 participants.

All analyses were performed using SPSS (version 27.0; IBM Corp). Baseline comparisons were made between the Calm intervention and waitlist control groups using independent samples 2-tailed *t* tests and Pearson chi-square analyses, as appropriate. As many baseline variables included numerous categories with potentially small cell sizes for chi-square analyses, omnibus baseline group comparisons were conducted with binary indicator variables collapsed across categories (White race vs non-White, presence vs absence of chronic sleep condition, etc). Similar comparisons were made for complete cases (ie, those with data available at all 5 time points from baseline to week 8) versus incomplete cases.

Inferential analyses were conducted using both complete-case (CC; analysis with data from participants completing all assessments) and intent-to-treat (ITT) approaches [50]. ITT analyses were conducted using the mixed models applied to all available data [51,52]. Mixed models were used to analyze group differences in outcomes over time. Mixed models are advantageous because they are well suited for longitudinal data with varying levels of missingness across participants. The models were estimated using maximum likelihood estimation procedures and assuming an autoregressive correlation structure. To allow for the evaluation of nonlinear change, time was treated as a factor, in which time points were individually dummy coded and compared with baseline. All models included dummy-coded indicators of gender; race; ethnicity; education; employee wage type (ie, hourly vs salaried worker); frontline-worker status (ie, working in retail stores or home services); and the presence of mental, physical, and sleep diagnoses as covariates. In addition, to account for potential effects of sleep coaching (ie, for the Calm intervention group, participants with elevated sleep disturbance who opted for sleep coaching) and completion of sleep diaries (ie, for the waitlist participants invited to sleep coaching after completing the waitlist period), we included 2 dummy-coded indicator variables reflecting enrollment in the sleep coaching program and participation in the program (ie, attended at least one coaching session). Of the 101 participants in the Calm intervention group who indicated interest in enrolling in the CCS program, 55 (54%) completed at least one coaching session. Among those who completed at least one coaching session, the median number of sessions attended was 4 (mean 4.1, SD 1.4). All models allowed for random effects of the person and the work site.

To estimate the effect size of the predictors, we calculated Cohen *d* by dividing the unstandardized regression coefficient by the SD of the outcome variable [53,54]. On the basis of recommendations in the study by Cohen [53], absolute *d* values near 0.30 and below were considered to reflect small effects, at or around 0.50 to reflect medium or moderate effects, and values near 0.80 or above were considered to reflect large effects.

Among participants assigned to the Calm group, descriptive statistics were generated to illustrate app use over time (ie, minutes per week using the Calm app and minutes per week using specific app components).

## Results

### Baseline Participant Characteristics

**Figure 1:**
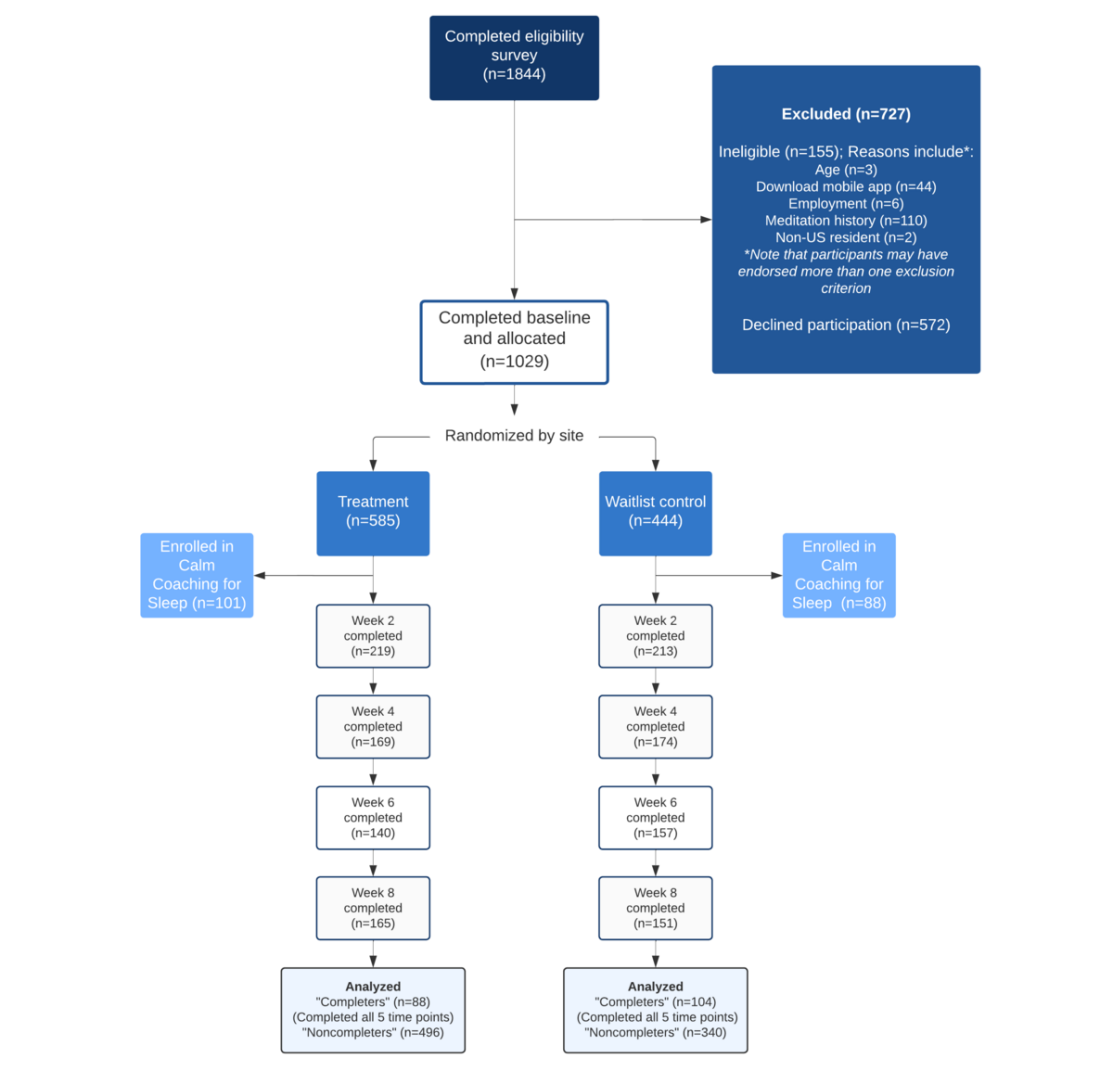
CONSORT (Consolidated Standards of Reporting Trials) diagram of study participation and analyzed data. *Note that participants may have been ineligible for more than one reason, all of which are reflected here. During the trial, participants could complete a subsequent assessment even if they missed a prior one.

Of the 1844 individuals screened for eligibility, 1689 (91.59%) were determined to be eligible and consented to participate. In total, 56.9% (585/1029) of participants were at sites randomized to the Calm intervention group, and 43.14% (444/1029) of participants were at sites randomized to the waitlist control. One participant did not provide sufficient data at any time point (including baseline) to be included in the analyses. Of participants who were randomized, 17.3% (101/444) in the Calm intervention group and 19.6% (87/444) in the control group opted for sleep coaching and sleep diaries. In total, 15% (88/585) of participants had CC data in the Calm intervention group (ie, had data available at all 5 time points), and 23.4% (104/444) of control participants had CC data. Participant flow through the full study is depicted in Figure 1.

Demographic and clinical characteristics of the participants in each group are described in Tables 1 and 2, along with group comparisons. The full sample was relatively evenly split between men (474/1024, 46.28%) and women (518/1024, 50.58%), and 3.12% (32/1024) of the participants identified with another gender. Most participants (803/1028, 78.11%) identified their race as White, and 15.46% (159/1028) identified with Hispanic or Latinx ethnicity. The groups did not differ at baseline in terms of sex, race, ethnicity, college education (yes or no), presence of a diagnosed sleep condition, or hourly versus salaried wage type (all *P*=.11). The waitlist group had a significantly higher proportion of individuals self-reporting a diagnosed mental health condition (*χ*^2^_1_=5.5; *P*=.02), and this variable was included as a covariate in the analyses (as planned) to control for this.

There were no significant baseline differences between groups on measures of depression, stress, insomnia, sleepiness, resilience, absenteeism, presenteeism, or work productivity impairment (Table 3). At baseline, waitlist participants had significantly higher anxiety (*P*=.04) and nonwork activity impairment (*P*=.046), and more frequent medical visits in the 4 weeks before study participation (*P*=.03). However, our modeling approach accounted for these baseline group differences using baseline scores as the reference for each subsequent time point evaluated. See Multimedia Appendix 1 for the group means across all time points.

With regard to comparisons of cases with complete data (ie, participants who provided survey data at all 5 study time points) and incomplete cases (ie, participants who completed surveys at fewer than 5 time points), complete cases were more likely to be people of color (*χ*^2^_1_=6.2; *P*=.01), more likely to be non-Hispanic (*χ*^2^_1_=4.6; *P*=.03), and less likely to have completed college education (*χ*^2^_1_=7.5; *P*=.01). Hourly workers were less likely than salaried workers to complete surveys at all 5 time points (*χ*^2^_1_=19.3; *P*<.001); however, among salaried employees, complete cases and incomplete cases did not differ with regard to salary (*t*_397_=0.13; *P*=.89), and among hourly workers, complete and incomplete cases did not significantly differ with regard to hourly wage (*t*_624_=1.26; *P*=.21).

**Table 1:** Demographic and employment characteristics of the sample^a^ (N=1029).

Characteristic				Waitlist, n (%)		Calm, n (%)		Chi-square (*df*)			*P* value	
**Gender**									0.1 (1)			.75
	Man			202 (45.7)		272 (46.7)						
	Woman			226 (51.1)		292 (50.1)						
	Other			14 (3.12)		18 (3.1)						
**Race**									2.4 (1)			.12
	American Indian or Alaskan native			12 (2.7)		16 (2.7)		0.001 (1)			.98	
	Asian or Asian American			16 (3.6)		33 (5.6)		2.3 (1)			.13	
	White or European American			340 (77.1)		463 (79.4)		0.8 (1)			.37	
	Black or African American			26 (5.9)		30 (5.1)		0.3 (1)			.60	
	Biracial or multiracial			27 (6.1)		26 (4.5)		1.4 (1)			.23	
	Other			33 (7.45)		20 (3.4)		8.4 (1)			.004	
**Ethnicity**									2.1 (1)			.14
	Non-Hispanic or Latino			367 (82.7)		503 (85.9)						
	Hispanic or Latino			77 (17.3)		82 (14)						
**Education level**									0.4 (1)			.53
	Less than high school			1 (0.2)		1 (0.1)						
	High school diploma			53 (11.9)		52 (89)						
	Some college			168 (37.9)		228 (39)						
	Associates or 2-year degree			70 (15.8)		60 (10.2)						
	Bachelor’s degree			112 (25.2)		175 (29.9)						
**Employee type**									18.0 (1)			<.001
	**Salaried (US $ per year)**			141 (31.8)		262 (44.8)						
		≤39.999	1 (0.7)		0 (0)							
		40,000-69,999	30 (21.4)		35 (13.5)							
		70,000-99,999	46 (32.9)		89 (34.4)							
		100,000-129,999	34 (24.3)		71 (27.4)							
		≥130,000	29 (20.7)		64 (24.7)							
	**Hourly (US $ per hour)**			303 (68.2)		323 (55.2)						
		<13.00	0 (0)		0 (0)							
		13.00-18.99	186 (61.4)		163 (50.5)							
		19.00-24.99	87 (28.7)		111 (34.3)							
		25.00-30.99	21 (6.9)		35 (10.8)							
		≥31.00	9 (2.9)		14 (4.3)							
**Employer insurance coverage**									0.2 (1)			.60
	Yes			316 (71.2)		425 (72.6)						
	No			128 (28.8)		160 (27.4)						
**Work setting**									74.3 (4)			<.001
	Market office			3 (0.7)		12 (2.1)						
	Store			328 (73.9)		306 (52.3)						
	Corporate			87 (19.6)		184 (31.5)						
	Home services			2 (0.5)		57 (9.7)						
	Other			24 (5.4)		26 (4.4)						

^a^Consistent with the operational definitions of demographic covariates in models of outcomes over time, chi-square tests reflect group comparisons of proportions of men and women, White and racial minority, Hispanic and non-Hispanic, completed and not completed college education, salaried and hourly employment status, and all work setting types.

**Table 2:** Health characteristics of the sample^a^ (N=1029).

Characteristic			Waitlist, n (%)	Calm, n (%)	Chi-square (*df*)		*P* value	
**Overall physical health**						7.7 (4)		.10
	Excellent		11 (2.4)	26 (4.4)				
	Very good		87 (19.5)	129 (22)				
	Good		197 (44.3)	261 (44.6)				
	Fair		120 (27)	147 (25.1)				
	Poor		29 (6.5)	22 (3.7)				
**Chronic health conditions**			254 (0.6)	320 (0.5)	1.3 (1)		.26	
	**Mental health condition**		175 (41.2)	193 (33.9)	5.5 (1)		.02	
		Depression	136 (32)	147 (25.8)	4.6 (1)		.03	
		PTSD^b^	32 (7.5)	35 (6.1)	0.7 (1)		.39	
		Anxiety disorder	139 (32.7)	139 (24.4)	8.3 (1)		.004	
	**Physical health condition**		144 (33.9)	171 (30.1)	1.7 (1)		.20	
		High blood pressure	71 (16.7)	68 (11.9)	4.6 (1)		.03	
		High cholesterol	40 (9.4)	41 (7.2)	1.6 (1)		.21	
		Diabetes	23 (5.4)	18 (3.1)	3.1 (1)		.08	
		Asthma or other lung disease	54 (12.7)	60 (10.5)	1.1 (1)		.29	
		Heart disease	5 (1.1)	9 (1.5)	0.3 (1)		.59	
		Arthritis or rheumatic disease	22 (5.1)	18 (3.1)	2.6 (1)		.11	
		Cancer	6 (1.4)	7 (1.2)	0.1 (1)		.80	
	Other chronic condition		48 (11.3)	65 (11.4)	0.004 (1)		.95	
**Sleep-related conditions**			96 (26.5)	121 (24.6)	0.4 (1)		.53	
	Insomnia		56 (0.1)	63 (0.1)	1.2 (1)		.27	
	Sleep apnea		33 (0.1)	50 (0.1)	0.3 (1)		.61	
	Narcolepsy		0 (0)	1 (0)	0.7 (1)		.39	
	Restless leg syndrome		18 (0.1)	18 (0.1)	0.9 (1)		.35	
	Somnambulism		1 (0.003)	2 (0)	0.1 (1)		.75	
	Night terrors		12 (0.03)	8 (0)	2.6 (1)		.12	
	Other sleep condition		3 (0.01)	10 (0.1)	2.0 (1)		.15	

^a^Consistent with operational definitions of health-related covariates in models of outcomes over time, chi-square tests reflect group comparisons of the proportions of the presence or absence of a chronic health condition and the presence or absence of a sleep-related condition.

^b^PTSD: posttraumatic stress disorder.

**Table 3:** Group differences in outcomes at baseline.

Measure	Waitlist		Calm		*t* test (*df*)	*P* value
	Values, N	Values, mean (SD)	Values, N	Values, mean (SD)		
ISI^a^	444	11.90 (5.87)	584	11.45 (5.65)	1.23 (1026)	.22
ESS^b^	443	7.10 (4.61)	582	7.26 (4.92)	−0.51 (1023)	.61
DASS-21^c^—depression	443	6.47 (5.12)	583	5.96 (4.93)	1.61 (1024)	.11
DASS-21—anxiety	443	4.96 (3.74)	584	4.49 (3.66)	2.02 (1025)	.04
DASS-21—stress	443	8.01 (4.29)	584	7.65 (4.08)	1.39 (1025)	.17
BRS^d^	443	3.29 (0.88)	583	3.31 (0.80)	−0.31 (1024)	.76
WPAI^e^—absenteeism	415	4.95 (14.35)	559	4.48 (14.52)	0.50 (972)	.62
WPAI—presenteeism	406	30.62 (26.14)	547	28.10 (25.69)	1.49 (951)	.14
WPAI—overall work impairment	404	33.08 (28.44)	546	30.30 (27.93)	1.51 (948)	.13
WPAI—activity impairment	438	35.87 (28.70)	574	32.33 (27.16)	2.00 (1010)	.046
Medical visits	444	0.79 (1.17)	584	0.64 (1.08)	2.20 (1026)	.03
Costs due to impaired productivity (US $)	404	334.81 (347.92)	545	354.84 (378.93)	−0.83 (947)	.41

^a^ISI: Insomnia Severity Index.

^b^ESS: Epworth Sleepiness Scale.

^c^DASS-21: Depression Stress Anxiety Scale 21-item.

^d^BRS: Brief Resilience Scale.

^e^WPAI: Work Productivity and Activity Impairment questionnaire. Medical visits were self-reported by employees indicating the number of times they visited a health care provider in the past 4 weeks. Cost due to impaired productivity was calculated based on self-reported pay and WPAI overall work impairment percentages; this metric is reported in US $.

### Calm App Effects on Mental Health, Productivity, and Related Outcomes

#### Mental Health

**Figure 2:**
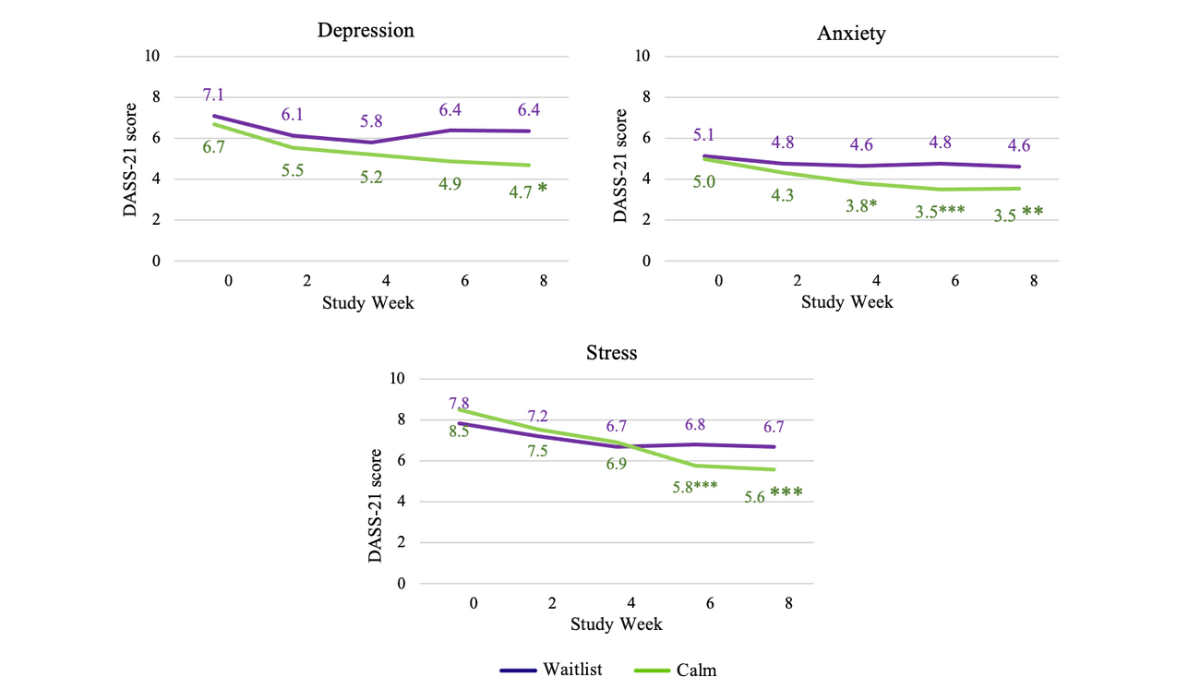
Estimated marginal means indicating group differences in changes in mental health outcomes over time among study completers. *P<.05; **P<.01; ***P<.001. DASS-21: Depression Anxiety Stress Scale-21 item.

In the CC analyses, participants at sites randomized to the Calm intervention group had significantly larger reductions in depression at week 8 than did participants at sites randomized to the waitlist control group (Figure 2; Table 4). In ITT analyses (ie, using all available data, inclusive of complete and incomplete cases [51,52]), participants in the Calm intervention group had significantly larger reductions in depression than participants in the waitlist control group at week 6 (Figure S1 in Multimedia Appendix 2). Among complete cases, Calm intervention group participants had significantly larger reductions in anxiety than the control group participants at weeks 4, 6, and 8; in ITT analyses, the effects of week 8 effects were retained. Similarly, complete cases in the Calm intervention group reported significantly larger reductions in stress at weeks 6 and 8 than did CC participants in the waitlist control group, whereas in the ITT analyses, employees using the Calm app had significantly greater reductions in stress at week 6 than those in the waitlist control group. Across all analyses, the effect sizes for changes in mental health indicated small to medium effects.

**Table 4:** Estimates of group differences in changes in mental health over time^a^.

Parameter		Complete cases				All available (ITT^b^)		
		*β* (SE; 95% CI)	*P* value	Cohen *d*	*β* (SE; 95% CI)		*P* value	Cohen *d*
**Depression**								
	Week 2× group	−0.18 (0.57; −1.32 to 0.95)	.75	−0.18	−0.34 (0.30; −0.93 to 0.25)		.26	−0.34
	Week 4× group	−0.19 (0.57; −1.31 to 0.93)	.74	−0.10	−0.02 (0.33; −0.67 to 0.62)		.94	−0.02
	Week 6× group	−1.09 (0.56; −2.18 to 0.01)	.052	−0.36	−0.73 (0.34; −1.41 to −0.06)		.03	−0.25
	Week 8× group	−1.27 (0.55; −2.35 to −0.19)	.02	−0.32	−0.66 (0.34; −1.32 to 0.001)		.05	−0.17
**Anxiety**								
	Week 2× group	−0.28 (0.38; −1.03 to 0.47)	.46	−0.28	−0.02 (0.23; −0.47 to 0.44)		.95	−0.02
	Week 4× group	−0.71 (0.34; −1.39 to −0.03)	.04	−0.36	−0.12 (0.24; −0.60 to 0.36)		.63	−0.07
	Week 6× group	−1.10 (0.37; −1.83 to −0.37)	.003	−0.37	−0.40 (0.27; −0.94 to 0.13)		.14	−0.14
	Week 8× group	−0.92 (0.36; −1.64 to −0.20)	.01	−0.23	−0.57 (0.24; −1.05 to −0.10)		.02	−0.15
**Stress**								
	Week 2× group	−0.36 (0.51; −1.37 to 0.65)	.48	−0.36	−0.14 (0.29; −0.71 to 0.42)		.62	−1.41
	Week 4× group	−0.47 (0.47; −1.39 to 0.46)	.32	−0.24	.08 (0.29; −0.49 to 0.65)		.79	−0.08
	Week 6× group	−1.70 (0.49; −2.66 to −0.74)	.001	−0.57	−0.77 (0.34; −1.45 to −0.09)		.03	0.02
	Week 8×group	−1.78 (0.50; −2.76 to −0.79)	<.001	−0.45	−0.51 (0.33; −1.16 to 0.13)		.12	−0.20

^a^Depression, anxiety, and stress were measured using the Depression Anxiety Stress Scale-21. Complete cases were defined as participants who provided survey data at all 5 time points. All available analyses included all data points from all participants, regardless of the number of survey time points completed. The baseline (week 0) was the reference group for all times by group interaction terms. For parameter estimates for the complete model, see Tables S1 and S2 in Multimedia Appendix 3.

^b^ITT: intent to treat.

#### Sleep

**Figure 3:**
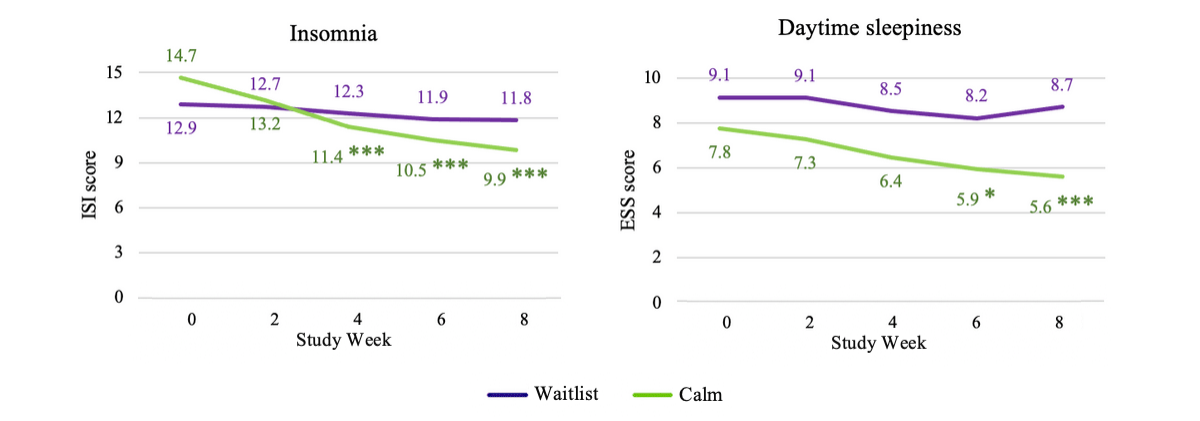
Estimated marginal means indicating group differences in changes in sleep outcomes over time among study completers. *P<.05; **P<.01; ***P<.001. ESS: Epworth Sleepiness Scale; ISI: Insomnia Severity Index.

In the CC analyses, participants using the Calm app had significantly larger reductions in insomnia symptoms at weeks 4, 6, and 8 than waitlist participants (Figure 3; Table 5). The results from the ITT analysis were similar, with participants using the Calm app reporting greater reductions in insomnia symptoms at weeks 2, 4, 6, and 8 (Figure S2 in Multimedia Appendix 2). Observed effects for insomnia were large in the CC analyses and medium in the ITT analyses. For both CC and ITT analyses, participants using the Calm app also reported greater reductions in daytime sleepiness than waitlist controls at weeks 6 and 8, with small to medium effect sizes observed in CC and small effect sizes observed in ITT analyses.

**Table 5:** Estimates of group differences in changes in sleep symptoms over time^a^.

Parameter		Complete cases				All available (ITT^b^)		
		*β* (SE; 95% CI)	*P* value	Cohen *d*	*β* (SE; 95% CI)		*P* value	Cohen *d*
**Insomnia symptoms**								
	Week 2× group	−1.37 (0.74; −2.84 to 0.09)	.07	−1.37	−0.78 (0.40; −1.57 to −0.005)		.049	−0.02
	Week 4× group	−2.65 (0.66; −3.95 to −1.35)	<.001	−1.33	−1.51 (0.39; −2.27 to −0.75)		<.001	−0.37
	Week 6× group	−3.18 (0.67; −4.49 to −1.87)	<.001	−1.06	−1.99 (0.43; −2.83 to −1.15)		<.001	−0.22
	Week 8× group	−3.74 (0.70; −5.13 to −2.36)	<.001	−0.94	−1.94 (0.48; −2.89 to −1.00)		<.001	1.08
**Daytime sleepiness**								
	Week 2× group	−0.47 (0.51; −1.49 to 0.54)	.36	−0.47	−0.53 (0.31; −1.15 to 0.08)		.09	−0.53
	Week 4× group	−0.70 (0.45; −1.60 to 0.19)	.12	−0.35	−0.57 (0.32; −1.21 to 0.06)		.08	−0.29
	Week 6× group	−0.91 (0.46; −1.81 to −0.01)	.047	−0.30	−0.77 (0.34; −1.44 to −0.10)		.02	−0.26
	Week 8×group	−1.73 (0.47; −2.66 to −0.79)	<.001	−0.43	−1.25 (0.34; −1.92 to −0.58)		<.001	−0.32

^a^Insomnia symptoms were measured using the Insomnia Severity Scale; daytime sleepiness was measured using the Epworth Sleepiness Scale. Complete cases were defined as participants who provided survey data at all 5 time points; all available analyses included all data points from all participants, regardless of the number of survey time points completed. The baseline (week 0) was the reference group for all times by group interaction terms. For the parameter estimates for the complete model, see Tables S3 and S4 in Multimedia Appendix 3.

^b^ITT: intent to treat.

#### Resilience

In the CC analysis, Calm intervention group participants had significantly greater (ie, more favorable) resilience scores (BRS) in weeks 4 and 8 (Table 6; small effect sizes), but there were no significant differences observed between groups at any time point in the ITT analysis.

**Table 6:** Estimates of group differences in changes in resilience over time^a^.

Parameter	Complete cases				All available (ITT^b^)		
	*β* (SE; 95% CI)	*P* value	Cohen *d*	*β* (SE; 95% CI)		*P* value	Cohen *d*
Week 2×group	.07 (0.08; −0.09 to 0.23)	.37	0.07	.01 (0.05; −0.09 to 0.11)		.85	0.01
Week 4×group	.20 (0.08; 0.04 to 0.37)	.02	0.10	.07 (0.06; −0.05 to 0.18)		.24	0.04
Week 6×group	.15 (0.08; −0.01 to 0.31)	.07	0.05	.05 (0.06; −0.07 to 0.16)		.40	0.02
Week 8×group	.21 (0.09; 0.04 to 0.38)	.02	0.05	.08 (0.06; −0.04 to 0.21)		.17	0.02

^a^Resilience was measured using the Brief Resilience Scale. Complete cases were defined as participants who provided survey data at all 5 time points; all available analyses included all data points from all participants, regardless of the number of survey time points completed. The baseline (week 0) was the reference group for all times by group interaction terms. For parameter estimates for the complete model, see Tables S5 and S6 in Multimedia Appendix 3.

^b^ITT: intent to treat.

#### Work Productivity and Impairment

**Figure 4:**
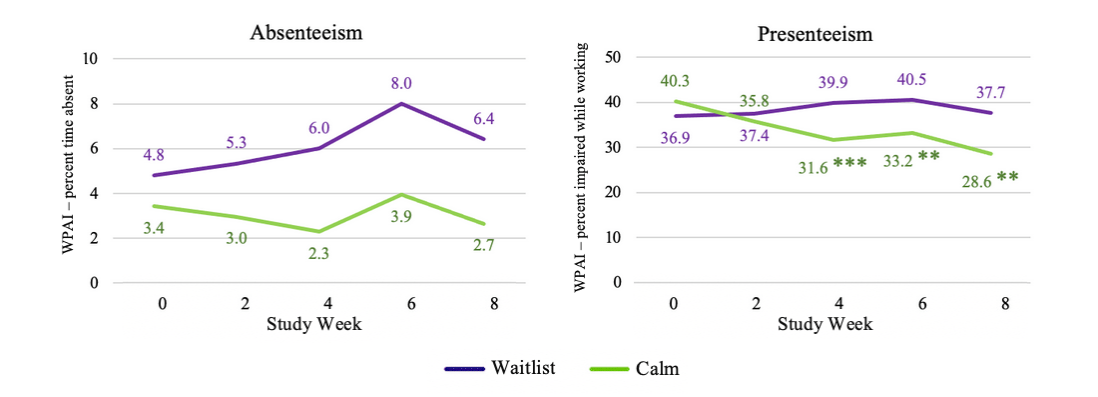
Estimated marginal means indicating group differences in absenteeism and presenteeism over time among study completers. *P<.05; **P<.01; ***P<.001. WPAI: work productivity and activity impairment.

**Figure 5:**
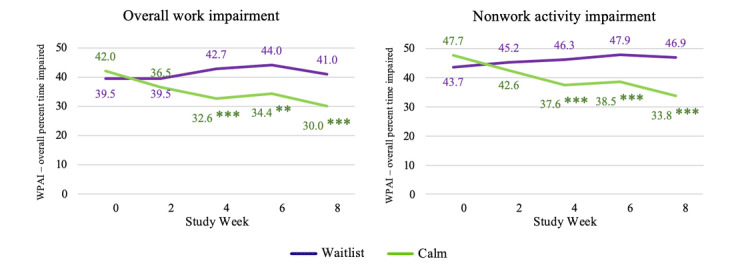
Estimated marginal means indicating group differences in changes in work and nonwork activity impairment over time among study completers. *P<.05; **P<.01; ***P<.001. WPAI: work productivity and activity impairment.

For absenteeism, no significant differences were observed between the groups at any time point in either the CC analysis or the ITT analysis (Figure 4; Table 7). For presenteeism, Calm intervention group participants were observed to have significantly lower impairment during work time than control participants at weeks 4, 6, and 8 for the CC analysis (Figure 4) but not for ITT. Small to medium effect sizes were observed for weeks 4, 6, and 8 in the CC analysis. In terms of overall work productivity impairment, participants assigned to the Calm intervention group had significantly lower impairment (ie, were more productive) at weeks 4, 6, and 8 in the CC analysis (Figure 5; medium effect sizes); the effects were not significant in ITT (small effect; Figure S3 in Multimedia Appendix 2). A significant benefit of the Calm app was observed on nonwork activity impairment at weeks 4, 6, and 8 in the CC analysis (Figure 5; medium effect sizes) and week 8 in the ITT analysis (small effect; Figure S4 in Multimedia Appendix 2).

**Table 7:** Estimates of group differences in changes in work productivity and activity impairment over timea.

Parameter		Complete case				All available (ITT^b^)		
		*β* (SE; 95% CI)	*P* value	Cohen *d*	*β* (SE; 95% CI)		*P* value	Cohen *d*
**Absenteeism**								
	Week 2× group	−0.99 (2.37; −5.66 to 3.67)	.67	−0.08	−0.19 (1.71; −3.56 to 3.18)		.91	−0.01
	Week 4× group	−2.35 (2.04; −6.37 to 1.67)	.25	−0.19	−0.10 (1.48; −3.01 to 2.80)		.95	−0.01
	Week 6× group	−2.69 (3.36; −9.34 to 3.95)	.42	−0.21	−1.01 (2.41; −5.76 to 3.74)		.67	−0.07
	Week 8× group	−2.40 (2.50; −7.34 to 2.53)	.34	−0.19	−0.16 (1.75; −3.61 to 3.29)		.93	−0.01
**Presenteeism**								
	Week 2× group	−4.90 (4.30; −13.36 to 3.57)	.26	−0.20	3.92 (2.53; −1.05 to 8.88)		.12	0.15
	Week 4× group	−11.59 (3.92; −19.32 to −3.86)	.004	−0.46	−2.42 (2.47; −7.28 to 2.43)		.33	−0.10
	Week 6× group	−10.58 (4.11; −18.69 to −2.46)	.01	−0.42	−2.39 (2.87; −8.03 to 3.26)		.41	−0.10
	Week 8×group	−12.35 (4.37; −20.97 to −3.72)	.01	−0.49	−2.44 (2.80; −7.94 to 3.06)		.38	−0.10
**Overall work impairment**								
	Week 2× group	−5.46 (4.46; −14.25 to 3.34)	.22	−0.21	4.32 (2.72; −1.02 to 9.66)		.11	0.15
	Week 4× group	−12.62 (4.24; −20.98 to −4.26)	.003	−0.47	−2.06 (2.72; −7.40 to 3.28)		.45	−0.08
	Week 6× group	−12.15 (4.38; −20.79 to −3.51)	.01	−0.45	−2.90 (3.06; −8.91 to 3.10)		.34	−0.11
	Week 8×group	−13.47 (4.61; −22.57 to −4.37)	.004	−0.51	−2.28 (2.99; −8.15 to 3.59)		.45	−0.09
**Nonwork activity impairment**								
	Week 2× group	−6.68 (4.25; −15.05 to 1.69)	.12	−0.24	2.78 (2.51; −2.15 to 7.71)		.27	0.10
	Week 4× group	−12.70 (3.66; −19.92 to −5.49)	.001	−0.45	−1.75 (2.36; −6.39 to 2.89)		.46	−0.06
	Week 6× group	−13.40 (4.01; −21.31 to −5.49)	.001	−0.47	−4.10 (2.89; −9.77 to 1.58)		.16	−0.15
	Week 8×group	−17.12 (4.03; −25.06 to −9.18)	<.001	−0.61	−5.64 (2.74; −11.01 to −0.26)		.04	−0.20

^a^All the outcomes were measured using the Work Productivity and Activity Impairment Questionnaire. Complete cases were defined as participants who provided survey data at all 5 time points; all available analyses included all data points from all participants, regardless of the number of survey time points completed. The baseline (week 0) was the reference group for all times by group interaction terms. For parameter estimates for the complete model, see Tables S7 and S8 in Multimedia Appendix 3.

^b^ITT: intent to treat.

#### Medical Care Visits

In both the CC and ITT analyses, participants randomized to the Calm intervention group had a significantly lower frequency of visits with a medical provider at week 8 than individuals in the waitlist control group (Table 8; small effect sizes in both models). No significant differences were observed at week 4.

**Table 8:** Estimates of group differences in changes in health care visits over timea.

Parameter			Complete cases				
			*β* (SE; 95% CI)		*P* value		Cohen *d*
**Complete cases**							
	Week 4×group	.02 (0.18; −0.34 to 0.37)		.93		0.01	
	Week 8×group	−1.05 (0.21; −1.46 to −0.65)		<.001		−0.26	
**All available (ITT** ^b^ **)**							
	Week 4×group	.15 (0.11; −0.05 to 0.36)		.14		0.08	
	Week 8×group	−0.93 (0.13; −1.19 to −0.68)		<.001		−0.23	

^a^Health care visits determined by responses to the question “How many times have you seen a medical provider in the last four weeks?” Complete cases were defined as participants who provided survey data at all 5 time points; all available analyses included all data points from all participants, regardless of the number of survey time points completed. The baseline (week 0) was the reference group for all times by group interaction terms. For parameter estimates for the complete model, see Tables S9 and S10 in Multimedia Appendix 3.

^b^ITT: intent to treat.

#### Productivity Cost Savings

**Figure 6:**
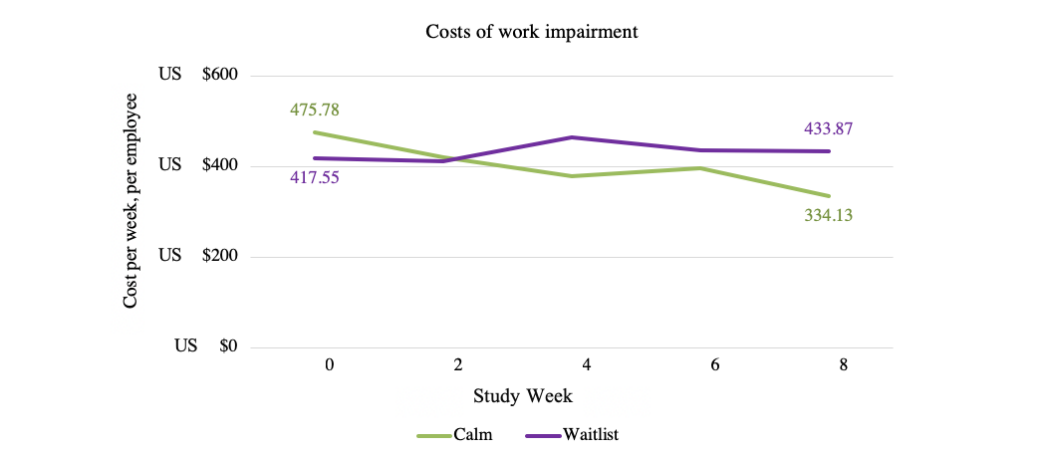
Estimated marginal means indicating group differences in costs of work impairment over time among study completers.

There was no significant effect of the Calm app on productivity costs in the ITT analysis. Significant productivity cost savings due to the Calm app were found at weeks 4 and 8 in the CC analysis, with medium effect sizes (Figure 6; Table 9). Among CC participants, the estimated average overall weekly productivity cost associated with work impairment (ie, absenteeism and presenteeism combined) for the Calm intervention group was US $334.13 (SE US $45.61) per employee per week at week 8, compared with US $475.78 (SE US $46.75) at baseline; for waitlist controls, costs associated with work impairment were estimated to be US $433.87.67 (SE US $55.47) at week 8 compared with US $417.55 (SE US $55.95) at baseline. This corresponded to a reduction in weekly costs by US $157.97 (SE US $58.37) per employee attributable to the Calm intervention over 8 weeks.

**Table 9:** Estimates of group differences in changes in work impairment costs over time^a^.

Parameter	Complete cases				All available (ITT^b^)		
	*β* (SE; 95% CI)	*P* value	Cohen *d*	*β* (SE; 95% CI)		*P* value	Cohen *d*
Week 2× group	−49.67 (51.33; −150.98 to 51.65)	.33	−0.15	52.43 (34.85; −16.05 to 120.92)		.13	0.15
Week 4× group	−141.83 (55.87; −251.97 to −31.70)	.01	−0.43	−33.30 (38.21; −108.43 to 41.82)		.38	−0.09
Week 6× group	−97.49 (54.62; −205.20 to 10.21)	.08	−0.29	−24.05 (43.61; −109.87 to 61.76)		.58	−0.07
Week 8×group	−155.66 (57.76; −269.66 to −41.66)	.01	−0.47	−28.41 (40.80; −108.64 to 51.82)		.49	−0.08

^a^Work impairment costs were calculated by multiplying an employee’s weekly pay by their overall work impairment percentage (ie, absenteeism and presenteeism). Complete cases were defined as participants who provided survey data at all 5 time points; all available analyses included all data points from all participants, regardless of the number of survey time points completed. The baseline (week 0) was the reference group for all times by group interaction terms. For parameter estimates for the complete model, see Tables S11 and S12 in Multimedia Appendix 3.

^b^ITT: intent to treat.

### Calm App Use

**Figure 7:**
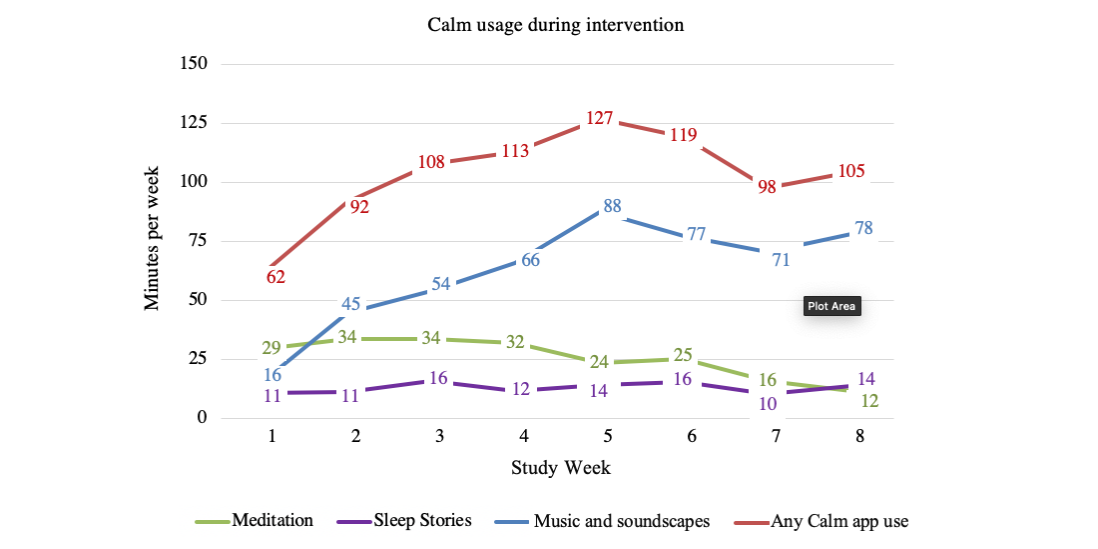
Average Calm app use per employee per week during the intervention period. This figure depicts the overall app use data (use of any component of the app), as well as the use of the most popular components. Less used components are not included in the figure such that the sum of the components presented does not encompass all app use.

Of the 585 employees randomized to the Calm intervention group, 265 (45.2%) downloaded the Calm app and used it at least once. On average, employees used the Calm app for 102.83 (SD 497.14) minutes per week (average sessions 5.88, SD 23.17). The most popular content was music, soundscapes, and sleep stories (Figure 7; Multimedia Appendix 4).

## Discussion

### Principal Findings

The purpose of this study was to evaluate the experimental effects of the Calm app on employee mental health (depression, anxiety, and stress), sleep, resilience, work productivity outcomes (absenteeism, presenteeism, overall work impairment, and overall activity impairment), and health care use (number of visits with a medical provider) in the workplace. This is the first study to evaluate the Calm app in a workplace setting, specifically with a focus on employee mental health and productivity. Results from a CC analysis (ie, participants completing assessments at all time points) indicated that the Calm app conferred significant benefits to employees in terms of mental health (depression, anxiety, and stress), sleep, resilience, several aspects of productivity (ie, presenteeism, overall work productivity impairment, and nonwork activity impairment), and the frequency of medical visits by the end of the study period. Overall, the effect size calculations indicated small to medium effects. Results from a more conservative analysis with all available data (including incomplete cases that did not provide data at all time points) showed a similar pattern of findings with significant effects observed for depression, anxiety, sleep, nonwork activity impairment, and medical visits.

### Mental Health, Sleep, and Resilience

Differences between the Calm group and waitlist control group were consistently observed by the end of the 8-week intervention for depression, anxiety, and stress, indicating that the beneficial effects of mindfulness training on mental health may be cumulative over time. The results align with prior findings, suggesting that mindfulness interventions provide benefits for anxiety and stress in the workplace and provide further support for their utility in improving depressive symptoms [18,20,22]. Furthermore, these results support the use of digital, app-based interventions as a viable and effective alternative to in-person approaches.

Employees at sites assigned to the Calm intervention group had significant improvements in sleep relative to waitlist control, with robust effects observed in both CC and ITT analyses. Thus, the Calm app provides benefits for mental health and sleep. These effects may have been synergistic, as sleep and mental health are known to have bidirectional effects on one another [55,56]. Indeed, prior work from our team has demonstrated that individuals with sleep disturbance experience improvements in both sleep quality and mental health because of the use of the Calm app [46,57]. In use analyses from this study, participants were observed to use a wide range of Calm app content, including but not limited to sleep stories and other content designed specifically to improve sleep. Although beyond the scope of this study, future work may explore whether engagement with a particular type of content is differentially associated with improvements in certain mental health and sleep constructs.

Relative to the waitlist control, significantly greater improvements in resilience were also observed in the Calm intervention group relative to waitlist control. This effect was only observed in the analysis of CC data and not when incomplete cases were included. Results from the CC analysis align with meta-analytic findings that mindfulness-based interventions in the workplace (inclusive of both in-person and digital delivery modes) improve resilience among employees across a range of employment settings [22].

In the CC analysis, significant reductions were observed in on-the-job work impairment due to physical or mental health concerns (presenteeism) in the Calm intervention group compared with the waitlist control group. However, there were no significant reductions in absenteeism (missed work time) in either the CC or ITT analyses. Thus, it appears that the Calm app may have been more beneficial in terms of improving employees’ ability to focus and stay present on the job versus preventing them from missing time from work entirely. These results are consistent with a handful of other studies that have suggested that mindfulness may pose greater benefits for presenteeism and overall productivity than absenteeism [25,26,58]. It is important to note that we also observed a relatively small degree of absenteeism at baseline (approximately 5%); thus, the results may reflect a floor effect with little room for improvement in a general sample. Furthermore, given that absenteeism is typically associated with more severe mental health problems, it is possible that employees require additional support beyond that of a stand-alone app (eg, brief, focused behavioral health coaching) to reduce the time missed when it is caused by mental health problems. Future studies are warranted to test the potential utility of additional support for those with higher levels of absenteeism (especially when absenteeism is attributable to chronic mental health problems).

We also observed significant reductions in the frequency of medical visits among Calm intervention group participants relative to waitlist controls. Given the strong associations among mental health, sleep, and health care use [11-13], it is possible that participants who experienced improvements in mental health because of using the Calm app also felt more able to cope with the stressors they encountered in daily life and work and consequently had a reduced need for medical care, including mental health–related visits. Although this area is less well studied, the findings of this study align with results from previous smaller studies of mindfulness-based interventions in the workplace, which have found that mental and physical health care use and costs are reduced among employees after receiving in-person mindfulness training [59,60].

A key question for employers is whether the benefits of the Calm app on mental health, sleep, and productivity also correspond to financial benefits in terms of cost savings for each employee. To our knowledge, this is the first study to evaluate the effects of a mindfulness app on workplace costs related to health-related work impairment. Although no significant effects were found in the ITT analysis, results from the CC analysis indicated that the Calm app reduced weekly costs by US $155.82 over 8 weeks of the intervention. This is consistent with a recently published study reporting that an employer-sponsored mental health program in the workplace was associated with a positive financial return on investment for employers across multiple worksites and industries [61]. However, this study estimated the cost for mental health services by employees, employers, or insurers and used arbitrary salaries for their analysis. Long-term studies are needed to understand how patterns of productivity cost savings evolve with longer-term employee use of the Calm app and how the results might apply to a broader population with more variable patterns of engagement.

### Strengths

This study builds upon the extant literature by improving our understanding of the concurrent effects of the Calm mindfulness app on both mental health outcomes and employee productivity in the workplace using a pragmatic implementation approach that maximizes the generalizability of findings. The Calm app was implemented with a large employer composed of hundreds of sites distributed geographically across the United States and included participants across a variety of income levels and educational backgrounds. The app was deployed entirely by the employer within its workforce, helping to provide pragmatically driven, real-world estimates of the potential reach and uptake of mindfulness apps in similar workplace settings. Participants were blinded to the Calm app brand and received very limited incentive for participating; thus, we anticipate that intervention uptake would be even higher in the future with brand recognition and more employee incentives for using the app as a part of a wellness program [62]. Furthermore, participants were instructed to use the Calm app autonomously and not specifically required to use certain features of the app, which is consistent with how any paying subscribers would engage with the app. Engagement with the app in this study is therefore representative of what employees would likely experience if the Calm app were offered as a component of employee wellness offerings. Finally, this study was strengthened by the frequent assessment of outcomes *throughout* the intervention period, allowing for a more precise evaluation of the timing at which mindfulness interventions begin to confer their benefits on employee mental health and productivity.

### Limitations

Despite this study’s strengths, its important limitations must be considered. First, relatively high attrition rates were observed in this study, especially compared with in-person mindfulness interventions in the workplace [27,63,64]. The most common self-reported reason for dropping out (of a total of 63 participants completing the dropout survey) was a lack of time (19/63, 30%) followed by a lack of motivation (11/63, 17%). This high attrition is attributable to two factors: (1) relative to in-person interventions, higher rates of attrition are consistently observed when evaluating digital behavioral health interventions, particularly in the context of pragmatic research where researcher contact does not serve as an incentive or reminder to use and engage with the intervention. Thus, while attrition may have attenuated the positive impact of the Calm app in this sample, the attrition experienced was not unique to this setting or specific intervention. High attrition also precluded us from randomizing participants with sleep disturbance to the sleep coaching intervention. However, in retrospect, one could argue that offering the sleep coaching program to all individuals within the Calm intervention group allowed for a more ecologically valid evaluation, as this is the approach an employer would likely take when the Calm app is offered alongside a more intensive intervention for individuals with elevated levels of distress. It is also important to note that results from an ITT analysis that included all available data from complete cases (those who completed all 5 study assessments) and incomplete cases (those who were lost to attrition or missed assessments) indicated less robust benefits of the Calm app. This is unsurprising given that those participants who started in the Calm intervention group but did not continue using the Calm app would be less likely to benefit. In part due to the sheer size and multisite nature of the workplace in which the Calm app was implemented, we encountered technical and implementation difficulties related to notifying employees of the availability of the study and in sending email reminders to participants to complete assessments. While a majority of the recruitment was conducted through the company’s human resources and via their internal email communications, the employer notified us of a potentially low email read rate for messages from their human resources department. This was particularly likely among the large portion of employees for whom regular email checking was not required for their job role (ie, storefront workers); this limited our reach with recruitment. Once individuals were enrolled in the study, we encountered an additional communication barrier, as the company’s internet security systems blocked a majority of the study’s communication efforts and data collection links or required employees to take extra steps to navigate around security filters. With this in mind, we asked participants to remember to open their emails and complete their assessments outside of their work site and on a personal device not owned by the company, which added an additional step for communication and limited our ability to reach all participants consistently. Implementation of workplace wellness programs is most successful when support for and communication about the intervention comes from within the organization and when messaging is consistent over time [65]. Third, despite music in the Calm app being the most popular content used on the app and the fact that employees used music in the Calm music for an average of 61.72 (SD 23.1) minutes per week, it is uncertain whether listening to music in the Calm app specifically led to improvements in mental health or productivity. Considering the popularity of music in the Calm app and many consumer-based mobile apps that offer similar music content, future studies are warranted to determine the effects of music on mental health and productivity. Furthermore, as is to be expected, we observed variable engagement across work sites, which could indicate other site-level characteristics (eg, workplace culture, networks of communication, and psychological safety within a team) that may have differentially influenced uptake across sites [66,67]. Future pragmatic trials implementing the Calm app in the workplace setting would benefit from additional consideration of these key contextual drivers of reach and uptake. Such trials should focus on testing strategies for engagement among employees with work roles that do not involve regular email communication (SMS text messaging, QR codes in break rooms, social media, etc). Furthermore, future studies should explore organization-level implementation strategies that can maximize the reach and uptake of the intervention.

### Conclusions

Commercial apps show promise as a feasible, scalable solution to reduce the burden of mental health problems on employees, improve productivity, and reduce costs for employers. Evidence suggests that mindfulness interventions (including those delivered via a smartphone app) may confer mental health benefits to employees, and the Calm app has been shown to improve mental health in a range of populations. This study adds to this evidence by suggesting that the Calm app improves employees’ mental health and productivity. Furthermore, to our knowledge, this is the first study to demonstrate support for productivity cost savings produced by a mindfulness app when implemented pragmatically in a workplace setting.

## Appendix

Multimedia Appendix 1 Group means on outcomes over time.

Multimedia Appendix 2 Change over time in intent-to-treat analysis (all available data).

Multimedia Appendix 3 Full treatment effects models.

Multimedia Appendix 4 Employee app use over time.

Multimedia Appendix 5 CONSORT (Consolidated Standards of Reporting Trials) eHealth checklist.

## Abbreviations

BRS: Brief Resilience Scale
CC: complete-case
DASS: Depression Anxiety Stress Scale
HCA: Human Capital Approach
ISI: Insomnia Severity Index
ITT: intent-to-treat
WPAI: Work Productivity and Activity Impairment
