# Supplementary material for: Testing the Pragmatic Effectiveness of a Consumer-Based Mindfulness Mobile App in the Workplace: Randomized Controlled Trial
Source: JMIR Mhealth Uhealth. 2022 Sep 28;10(9):e38903. doi: 10.2196/38903 (PMC9557765; doi:10.2196/38903)
Supplement: Multimedia Appendix 1 [file mhealth_v10i9e38903_app1.pdf]

Table S1.1. Mean scores on outcome measures over time, by group (all available data)

Outcome	Group	Week 0			Week 2			Week 4			Week 6			Week 8		
		M	*SD*	*N*	*M*	*SD*	*N*	*M*	*SD*	*N*	*M*	*SD*	*N*	*M*	*SD*	*N*
Insomnia symptoms	Waitlist	11.90	5.87	444	11.43	5.68	230	10.93	5.99	182	11.23	6.20	164	11.09	6.58	157
	Calm	11.45	5.65	584	10.51	5.56	235	9.16	5.34	180	8.46	5.92	144	8.56	5.81	203
Daytime sleepiness	Waitlist	7.10	4.61	443	7.33	4.46	220	6.71	4.40	177	6.41	4.61	160	6.62	4.81	154
	Calm	7.26	4.92	582	6.83	4.26	225	6.44	4.76	172	5.55	4.36	141	5.77	4.69	196
Depression	Waitlist	6.47	5.12	443	5.42	4.69	224	4.80	4.72	180	5.34	5.42	161	5.13	5.22	156
	Calm	5.96	4.93	583	4.34	4.02	231	4.19	4.41	176	3.57	3.80	141	3.91	4.25	201
Anxiety	Waitlist	4.96	3.74	443	4.08	3.70	224	3.79	3.54	180	3.93	3.94	161	3.86	3.95	156
	Calm	4.49	3.66	584	3.37	3.39	231	3.26	3.62	176	2.72	3.30	141	2.85	3.21	201
Stress	Waitlist	8.01	4.29	443	7.32	4.32	224	6.79	4.18	180	6.73	4.66	161	6.70	5.08	156
	Calm	7.65	4.08	584	6.51	3.59	231	6.29	3.66	176	5.08	3.68	141	5.40	3.61	201
Resilience	Waitlist	3.29	0.88	443	3.39	0.87	223	3.43	0.96	178	3.43	1.04	160	3.43	0.99	155
	Calm	3.31	0.80	583	3.41	0.79	229	3.47	0.78	172	3.49	0.80	141	3.51	0.78	199
Absenteeism	Waitlist	4.95	14.34	415	4.41	15.43	203	3.63	11.53	166	5.04	17.89	139	4.61	15.59	136
	Calm	4.48	14.52	559	4.08	15.37	201	3.15	11.25	158	3.19	11.84	124	2.53	10.08	175
Presenteeism	Waitlist	30.62	26.14	406	25.00	25.99	200	27.33	25.12	161	27.01	26.10	134	29.33	27.10	135
	Calm	28.10	25.69	547	25.95	25.07	195	21.51	22.95	152	22.46	25.40	122	21.33	22.54	173
Work impairment	Waitlist	33.08	28.44	404	26.87	28.25	202	28.87	26.37	161	30.13	29.20	138	31.53	28.94	136
	Calm	30.30	27.93	546	27.87	26.94	197	23.09	24.80	153	23.41	26.14	120	22.81	24.08	174

Activity impairment	Waitlist	35.87	28.69	438	32.10	29.59	214	30.68	28.46	176	32.83	31.32	159	34.90	30.91	153
	Calm	32.33	27.16	574	31.80	27.74	217	25.92	25.08	169	24.47	26.76	141	24.18	26.12	194
Medical care visits	Waitlist	0.79	1.17	444				0.57	0.94	184				1.83	1.38	159
	Calm	0.64	1.08	584				0.54	0.97	183				0.65	1.11	206

Table S1.2. Mean scores on outcome measures over time, by group (complete cases only)

		Week 0			Week 2			Week 4			Week 6			Week 8		
		M	*SD*	*N*	*M*	*SD*	*N*	*M*	*SD*	*N*	*M*	*SD*	*N*	*M*	*SD*	*N*
Insomnia symptoms	Waitlist	4.29	3.64	104	3.83	3.77	104	3.70	3.60	104	3.68	3.89	104	3.67	4.02	104
	Calm	3.70	3.14	88	2.93	3.32	87	2.55	2.92	88	2.30	3.02	88	2.32	2.85	88
Daytime sleepiness	Waitlist	7.67	4.61	104	7.13	4.64	104	6.74	4.40	104	6.55	4.71	104	6.67	5.35	104
	Calm	7.47	3.50	88	6.52	3.57	87	5.76	3.20	88	4.77	3.10	88	4.55	2.98	88
Depression	Waitlist	11.28	5.86	104	11.20	5.79	104	10.74	5.84	104	10.54	5.98	104	10.45	6.55	104
	Calm	11.52	5.30	88	10.32	5.31	88	8.83	4.98	88	7.88	4.91	88	7.24	5.33	88
Anxiety	Waitlist	7.16	4.70	104	7.36	4.51	104	6.86	4.48	104	6.43	4.69	104	6.89	5.01	104
	Calm	6.97	4.58	88	6.52	3.97	88	5.85	4.19	88	5.18	4.10	88	4.95	4.19	88
Stress	Waitlist	6.19	5.65	104	5.32	5.02	104	4.94	5.09	104	5.19	5.43	104	5.28	5.72	104
	Calm	5.22	3.85	88	4.11	3.70	87	3.80	4.07	88	3.43	3.56	88	3.33	3.91	88
Resilience	Waitlist	3.30	0.99	104	3.34	1.00	104	3.36	1.02	104	3.38	1.02	104	3.42	1.06	104
	Calm	3.24	0.74	88	3.35	0.72	88	3.51	0.77	88	3.46	0.75	88	3.55	0.76	88
Absenteeism	Waitlist	3.25	9.82	102	3.54	12.02	97	4.21	12.99	101	5.23	18.44	91	5.19	17.81	94
	Calm	2.55	11.37	86	1.97	9.00	77	1.59	5.50	82	2.97	11.95	78	1.58	6.24	84
Presenteeism	Waitlist	27.04	24.71	98	26.60	27.31	97	28.27	26.09	98	27.73	27.32	88	27.74	27.39	93
	Calm	29.02	24.53	82	23.90	23.91	77	22.05	22.93	78	23.12	23.80	77	18.07	20.57	83
Work impairment	Waitlist	28.93	26.14	98	27.82	28.41	97	30.03	27.29	98	31.03	30.16	91	30.09	29.71	94
	Calm	30.57	25.90	83	24.38	24.29	77	22.84	23.86	78	23.94	24.64	75	19.08	21.42	83

Activity impairment	Waitlist	31.92	28.08	104	32.50	31.37	104	32.79	28.78	104	33.37	32.13	104	34.13	32.16	104
	Calm	33.41	24.02	88	28.30	27.72	88	25.11	24.54	88	23.30	24.76	88	20.00	22.15	87
Medical care visits	Waitlist	0.81	1.06	104				0.62	1.02	104				1.84	1.39	104
	Calm	0.68	1.12	88				0.55	0.90	88				0.66	1.13	88
