# Supplementary material for: Testing the Pragmatic Effectiveness of a Consumer-Based Mindfulness Mobile App in the Workplace: Randomized Controlled Trial
Source: JMIR Mhealth Uhealth. 2022 Sep 28;10(9):e38903. doi: 10.2196/38903 (PMC9557765; doi:10.2196/38903)
Supplement: Multimedia Appendix 2 [file mhealth_v10i9e38903_app2.pdf]

Figure S2.1. Estimated marginal means indicating group differences in changes in mental health outcomes over time from mixed models including all available data.

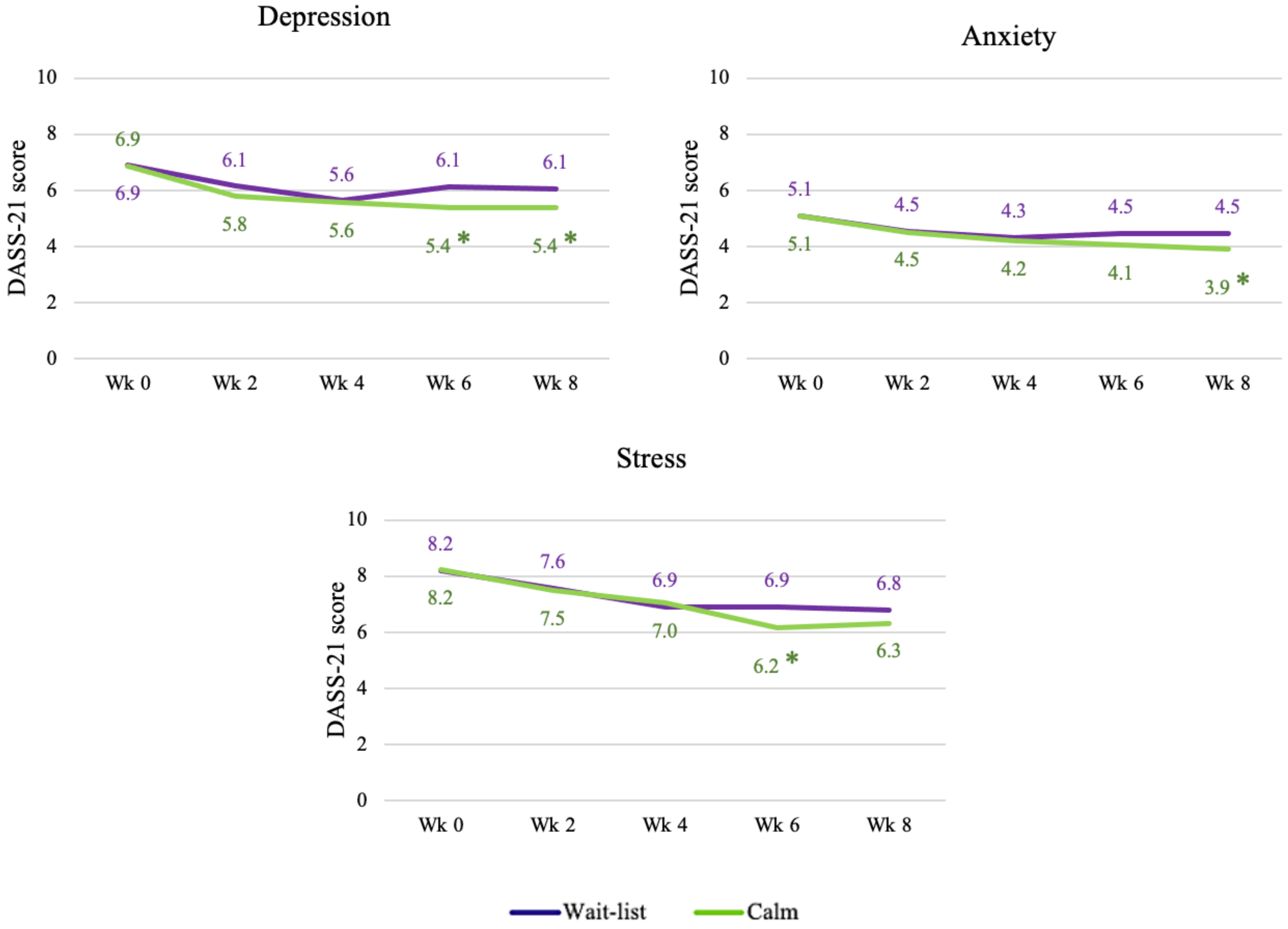

*p<.05; **p<.01; ***p<.001
Note. DASS-21 = Depression Anxiety Stress Scale

Figure S2.2. Estimated marginal means indicating group differences in changes in sleep outcomes over time from mixed models including all available data.

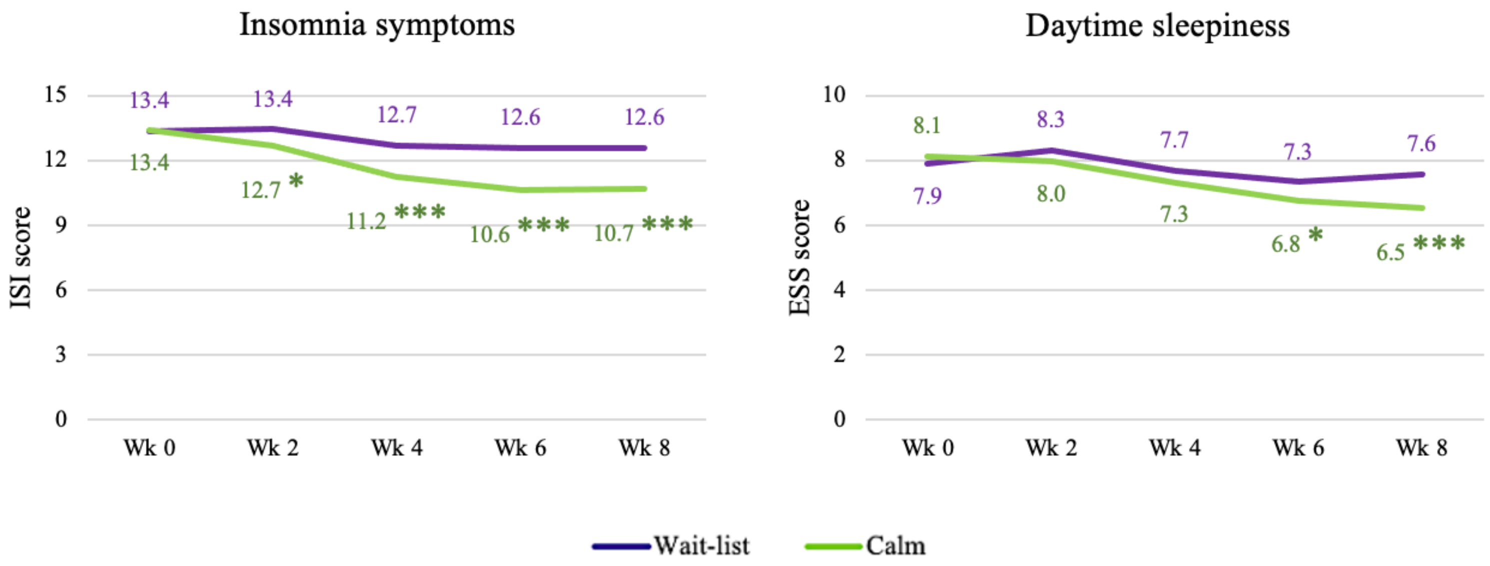

**p*<.05; ***p*<.01; ****p*<.001

Note. ISI = Insomnia Severity Index; ESS = Epworth Sleepiness Scale

Figure S2.3. Estimated marginal means indicating group differences in absenteeism and presenteeism over time from mixed models including all available data.

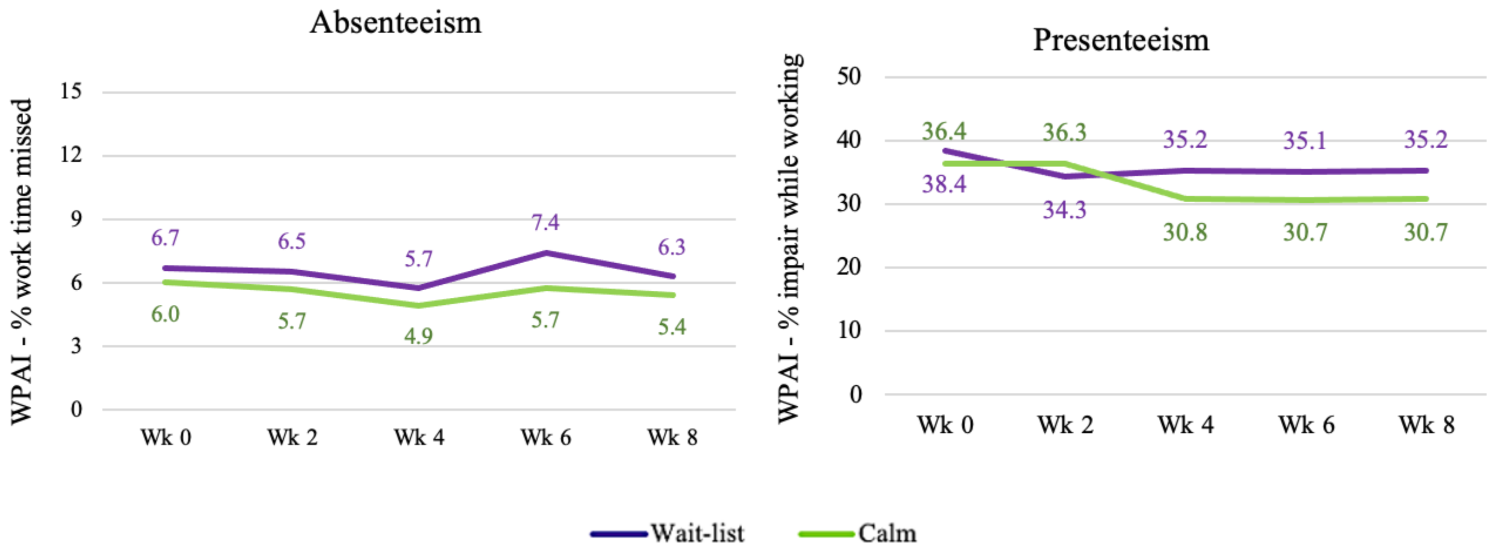

**p*<.05; ***p*<.01; ****p*<.001

Note. WPAI = Work Productivity and Activity Impairment Scale

Figure S2.4. Estimated marginal means indicating group differences in changes in work and non-work activity impairment over time from mixed models including all available data.

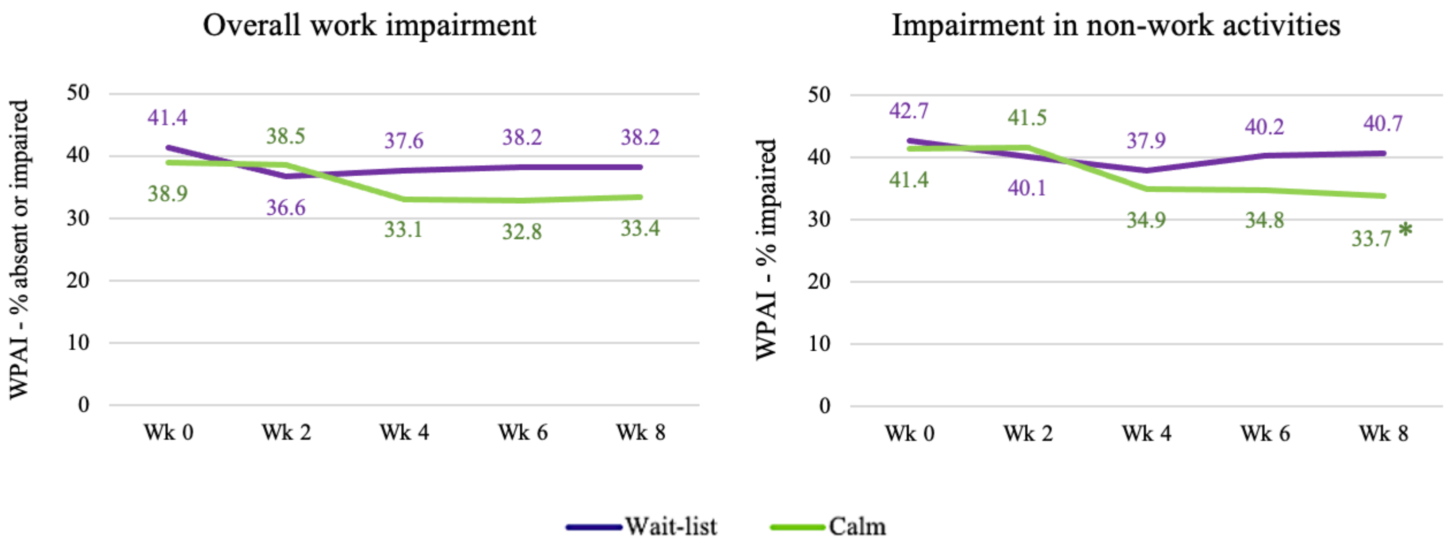

*p<.05; **p<.01; ***p<.001

Note. WPAI = Work Productivity and Activity Impairment Scale
