# Supplementary material for: Testing the Pragmatic Effectiveness of a Consumer-Based Mindfulness Mobile App in the Workplace: Randomized Controlled Trial
Source: JMIR Mhealth Uhealth. 2022 Sep 28;10(9):e38903. doi: 10.2196/38903 (PMC9557765; doi:10.2196/38903)
Supplement: Multimedia Appendix 3 [file mhealth_v10i9e38903_app3.pdf]

Table S3.1 Estimates of group differences in changes in mental health over time (all available data).

Parameter	Coefficient	*SE*	*p*	95% CI LL	95% CI UL	*d*
**Depression** (*N*=1026)						
Intercept	3.72	1.09	<.001	1.59	5.85	--
Gender=female	-0.77	0.30	.01	-1.36	-0.18	-1.54
Racial minority	0.72	0.36	.04	0.02	1.42	1.66
College education	-1.15	0.31	<.001	-1.76	-0.55	-2.30
Hourly worker	1.71	0.35	<.001	1.01	2.40	3.50
Frontline worker	-2.22	1.38	.11	-4.94	0.50	-4.73
Mental health dx	3.32	0.34	<.001	2.66	3.98	6.87
Sleep dx	0.61	0.36	.09	-0.09	1.31	1.40
CCS enrolled	0.59	0.43	.17	-0.26	1.44	1.53
CCS attended	0.07	0.70	.92	-1.31	1.45	0.31
Group=Calm	0.04	0.33	.91	-0.62	0.69	0.08
Week 2	-0.75	0.22	<.001	-1.18	-0.32	-1.87
Week 4	-1.26	0.24	<.001	-1.73	-0.80	-3.15
Week 6	-0.76	0.25	<.001	-1.24	-0.27	-1.90
Week 8	-0.83	0.25	<.001	-1.33	-0.33	-2.07
Week 2 * Group	-0.34	0.30	.26	-0.94	0.25	-0.34
Week 4 * Group	-0.03	0.33	.93	-0.67	0.62	-0.02
Week 6 * Group	-0.74	0.34	.03	-1.41	-0.06	-0.25
Week 8 * Group	-0.67	0.34	.048	-1.33	-0.01	-0.17
**Anxiety** (*N*=1027)						
Intercept	0.79	0.83	.34	-0.84	2.41	--
Gender=female	0.44	0.23	.054	-0.01	0.89	0.88
Racial minority	0.32	0.27	.24	-0.21	0.85	0.74
College education	-0.72	0.23	<.001	-1.18	-0.26	-1.44
Hourly worker	1.19	0.27	<.001	0.66	1.72	2.44
Frontline worker	0.13	1.05	.90	-1.94	2.19	0.28
Mental health dx	2.17	0.26	<.001	1.67	2.67	4.49
Sleep dx	1.09	0.27	<.001	0.55	1.62	2.50
CCS enrolled	0.69	0.33	.04	0.04	1.33	1.78
CCS attended	0.58	0.54	.28	-0.47	1.64	2.58
Group=Calm	0.02	0.25	.94	-0.48	0.52	0.04
Week 2	-0.54	0.17	<.001	-0.87	-0.20	-1.35
Week 4	-0.76	0.18	<.001	-1.11	-0.41	-1.90
Week 6	-0.61	0.19	<.001	-0.99	-0.23	-1.52
Week 8	-0.61	0.18	<.001	-0.97	-0.24	-1.52
Week 2 * Group	-0.02	0.23	.92	-0.48	0.43	-0.02
Week 4 * Group	-0.13	0.24	.60	-0.61	0.35	-0.07
Week 6 * Group	-0.41	0.27	.14	-0.94	0.13	-0.14

Week 8 * Group	-0.58	0.24	.02	-1.06	-0.10	-0.15
**Stress** (*N*=1027)						
Intercept	3.19	0.95	<.001	1.33	5.05	--
Gender=female	0.70	0.26	.01	0.18	1.21	1.40
Racial minority	0.38	0.31	.23	-0.23	0.99	0.87
College education	-1.05	0.27	<.001	-1.57	-0.52	-0.40
Hourly worker	0.96	0.31	<.001	0.35	1.57	-2.15
Frontline worker	-0.09	1.21	.94	-2.46	2.28	2.05
Mental health dx	2.70	0.29	<.001	2.13	3.28	-0.19
Sleep dx	0.71	0.31	.02	0.10	1.32	6.19
CCS enrolled	1.49	0.38	<.001	0.75	2.23	1.84
CCS attended	1.00	0.61	.10	-0.21	2.21	6.62
Group=Calm	0.00	0.29	.99	-0.57	0.56	2.02
Week 2	-0.61	0.21	<.001	-1.03	-0.20	0.00
Week 4	-1.28	0.21	<.001	-1.69	-0.87	-1.52
Week 6	-1.30	0.25	<.001	-1.78	-0.82	-3.20
Week 8	-1.40	0.25	<.001	-1.89	-0.91	-3.25
Week 2 * Group	-0.15	0.29	.60	-0.72	0.41	-1.41
Week 4 * Group	0.07	0.29	.81	-0.50	0.64	-0.08
Week 6 * Group	-0.78	0.34	.02	-1.45	-0.10	0.02
Week 8 * Group	-0.52	0.33	.11	-1.17	0.12	-0.20

Note. SE = standard error; *p* = *p*-value; 95% CI LL and UL = 95% confidence interval lower limit and upper limit; *d* = Cohens *d*; CCS=Calm Coaching for Sleep. Depression, anxiety, and stress were measured using the Depression Anxiety Stress Scale-21. Baseline (week 0) was the reference group for all time by group interaction terms.

Table S3.2. Estimates of group differences in changes in mental health over time (complete cases).

Parameter	Coefficient	*SE*	*p*	95% CI LL	95% CI UL	*d*
**Depression** (*N*=192)						
Intercept	2.81	2.22	.21	-1.58	7.21	--
Gender=female	-1.65	0.66	.01	-2.96	-0.35	-3.31
Racial minority	1.47	0.84	.08	-0.20	3.14	3.80
College education	-1.31	0.68	.055	-2.64	0.03	-2.67
Hourly worker	1.35	0.82	.10	-0.27	2.97	2.70
Frontline worker	1.14	4.09	.78	-6.94	9.22	2.31
Mental health dx	3.51	0.76	<.001	2.02	5.00	7.38
Sleep dx	0.23	0.76	.76	-1.26	1.72	0.51
CCS enrolled	0.79	0.83	.34	-0.85	2.43	1.65
CCS attended	0.34	1.20	.78	-2.04	2.71	0.97
Group=Calm	-0.49	0.90	.59	-2.26	1.29	-0.98
Week 2	-0.95	0.40	.02	-1.73	-0.16	-2.37
Week 4	-1.28	0.39	<.001	-2.06	-0.51	-3.20
Week 6	-0.72	0.39	.06	-1.48	0.04	-1.80
Week 8	-0.73	0.38	.06	-1.48	0.02	-1.82
Week 2 * Group	-0.18	0.58	.75	-1.32	0.95	-0.18
Week 4 * Group	-0.19	0.57	.74	-1.31	0.93	-0.10
Week 6 * Group	-1.09	0.56	.052	-2.18	0.01	-0.36
Week 8 * Group	-1.27	0.55	.02	-2.35	-0.19	-0.32
**Anxiety** (*N*=192)						
Intercept	0.75	1.57	.64	-2.35	3.85	--
Gender=female	-0.78	0.47	.10	-1.70	0.15	-1.57
Racial minority	1.62	0.60	.01	0.44	2.80	4.19
College education	-1.06	0.48	.03	-2.01	-0.12	-2.16
Hourly worker	1.55	0.58	.01	0.40	2.69	3.10
Frontline worker	0.26	2.89	.93	-5.46	5.97	0.53
Mental health dx	2.02	0.53	<.001	0.96	3.08	4.25
Sleep dx	0.54	0.53	.32	-0.52	1.59	1.19
CCS enrolled	0.03	0.59	.96	-1.13	1.19	0.06
CCS attended	-0.21	0.85	.80	-1.90	1.47	-0.60
Group=Calm	-0.43	0.61	.48	-1.64	0.77	-0.86
Week 2	-0.37	0.26	.16	-0.89	0.14	-0.92
Week 4	-0.49	0.24	.04	-0.96	-0.02	-1.22
Week 6	-0.37	0.26	.15	-0.88	0.14	-0.92
Week 8	-0.53	0.25	.04	-1.02	-0.03	-1.32
Week 2 * Group	-0.28	0.38	.46	-1.03	0.46	-0.28
Week 4 * Group	-0.71	0.34	.04	-1.38	-0.03	-0.36
Week 6 * Group	-1.10	0.37	<.001	-1.83	-0.37	-0.37

Week 8 * Group	-0.92	0.36	.01	-1.64	-0.20	-0.23
**Stress** (*N*=192)						
Intercept	1.97	2.00	.33	-1.98	5.92	--
Gender=female	-0.11	0.59	.86	-1.28	1.07	-0.22
Racial minority	1.05	0.76	.17	-0.45	2.56	2.71
College education	-1.28	0.61	.04	-2.49	-0.08	-2.61
Hourly worker	1.29	0.74	.08	-0.17	2.75	2.58
Frontline worker	1.52	3.68	.68	-5.75	8.80	3.08
Mental health dx	2.71	0.68	<.001	1.36	4.05	5.70
Sleep dx	0.60	0.68	.38	-0.74	1.94	1.32
CCS enrolled	1.41	0.75	.06	-0.07	2.89	2.94
CCS attended	1.43	1.08	.19	-0.71	3.57	4.10
Group=Calm	0.50	0.79	.52	-1.05	2.06	1.00
Week 2	-0.62	0.36	.08	-1.32	0.09	-1.55
Week 4	-1.13	0.32	<.001	-1.77	-0.49	-2.82
Week 6	-1.04	0.34	<.001	-1.70	-0.37	-2.60
Week 8	-1.14	0.35	<.001	-1.83	-0.46	-2.85
Week 2 * Group	-0.36	0.51	.48	-1.37	0.65	-0.36
Week 4 * Group	-0.47	0.47	.32	-1.39	0.45	-0.24
Week 6 * Group	-1.70	0.49	<.001	-2.66	-0.74	-0.57
Week 8 * Group	-1.78	0.50	<.001	-2.76	-0.79	-0.45

Note. SE = standard error; *p* = *p*-value; 95% CI LL and UL = 95% confidence interval lower limit and upper limit; *d* = Cohens *d*; CCS=Calm Coaching for Sleep. Depression, anxiety, and stress were measured using the Depression Anxiety Stress Scale-21. Complete cases were defined as participants who provided survey data at all five time points. Baseline (week 0) was the reference group for all time by group interaction terms.

Table S3.3. Estimates of group differences in changes in sleep symptoms over time (all available data).

Parameter	Coefficient	*SE*	*p*	95% CI LL	95% CI UL	*d*
**Insomnia symptoms** (*N*=1028)						
Intercept	8.84	0.59	<.001	7.68	9.99	--
Gender=female	0.63	0.37	.09	-0.09	1.36	1.26
Racial minority	1.12	0.41	.01	0.31	1.93	2.58
College education	-1.08	0.37	.004	-1.82	-0.35	-2.16
Hourly worker	0.80	0.42	.06	-0.04	1.63	1.64
Frontline worker	0.45	0.44	.31	-0.42	1.31	0.96
Mental health dx	2.12	0.41	<.001	1.31	2.94	4.39
Sleep dx	2.88	0.44	<.001	2.03	3.74	6.60
CCS enrolled	3.23	0.53	<.001	2.19	4.27	8.35
CCS attended	-1.07	0.87	.22	-2.77	0.63	-4.76
Group=Calm	0.04	0.40	.93	-0.75	0.82	0.08
Week 2	0.06	0.29	.83	-0.51	0.63	0.15
Week 4	-0.68	0.28	.02	-1.23	-0.13	-1.70
Week 6	-0.81	0.31	.01	-1.41	-0.20	-2.02
Week 8	-0.79	0.37	.03	-1.51	-0.08	-1.97
Week 2 * Group	-0.78	0.40	.049	-1.57	-0.005	-0.79
Week 4 * Group	-1.51	0.39	<.001	-2.27	-0.75	-0.76
Week 6 * Group	-1.99	0.43	<.001	-2.83	-1.15	-0.67
Week 8 * Group	-1.94	0.48	<.001	-2.89	-1.00	-0.49
**Daytime sleepiness** (*N*=1025)						
Intercept	5.52	1.20	<.001	3.17	7.87	--
Gender=female	0.26	0.33	.43	-0.39	0.91	0.52
Racial minority	0.11	0.39	.78	-0.66	0.88	0.25
College education	-0.59	0.34	.08	-1.25	0.07	-1.18
Hourly worker	0.33	0.39	.40	-0.44	1.10	0.68
Frontline worker	-0.59	1.52	.70	-3.56	2.39	-1.26
Mental health dx	0.72	0.37	.052	-0.01	1.45	1.49
Sleep dx	1.50	0.39	<.001	0.73	2.27	3.44
CCS enrolled	0.71	0.48	.14	-0.23	1.64	1.84
CCS attended	-0.27	0.78	.73	-1.80	1.27	-1.20
Group=Calm	0.32	0.36	.37	-0.38	1.03	0.65
Week 2	0.40	0.23	.08	-0.05	0.85	1.00
Week 4	-0.21	0.23	.36	-0.67	0.25	-0.52
Week 6	-0.57	0.24	.02	-1.05	-0.09	-1.42
Week 8	-0.32	0.26	.22	-0.83	0.19	-0.80
Week 2 * Group	-0.53	0.31	.09	-1.15	0.08	-0.53
Week 4 * Group	-0.58	0.32	.08	-1.21	0.06	-0.29
Week 6 * Group	-0.77	0.34	.02	-1.44	-0.10	-0.26

Week 8 * Group	-1.25	0.34	<.001	-1.93	-0.58	-0.32

SE = standard error; *p* = *p*-value; 95% CI LL and UL = 95% confidence interval lower limit and upper limit; *d* = Cohens *d*; CCS=Calm Coaching for Sleep. Insomnia symptoms were measured using the Insomnia Severity Scale; daytime sleepiness was measured using the Epworth Sleepiness Scale. Baseline (week 0) was the reference group for all time by group interaction terms.

Table S3.4. Estimates of group differences in changes in sleep symptoms over time (complete cases).

Parameter	Coefficient	*SE*	*p*	95% CI LL	95% CI UL	*d*
**Insomnia symptoms** (*N*=192)						
Intercept	5.52	2.68	.04	0.23	10.82	--
Gender=female	-0.93	0.80	.25	-2.50	0.64	-1.87
Racial minority	1.84	1.02	.07	-0.18	3.85	4.75
College education	-1.46	0.81	.08	-3.07	0.15	-2.97
Hourly worker	0.89	0.99	.37	-1.06	2.84	1.78
Frontline worker	1.06	4.93	.83	-8.67	10.80	2.15
Mental health dx	3.27	0.91	<.001	1.47	5.07	6.87
Sleep dx	2.56	0.91	.01	0.76	4.35	5.62
CCS enrolled	3.59	1.00	<.001	1.61	5.56	7.49
CCS attended	1.69	1.45	.25	-1.18	4.55	4.84
Group=Calm	1.35	1.08	.21	-0.78	3.49	2.70
Week 2	-0.14	0.51	.78	-1.16	0.87	-0.35
Week 4	-0.62	0.46	.18	-1.52	0.29	-1.55
Week 6	-1.00	0.46	.03	-1.91	-0.09	-2.50
Week 8	-1.06	0.49	.03	-2.02	-0.11	-2.65
Week 2 * Group	-1.37	0.74	.07	-2.84	0.09	-1.37
Week 4 * Group	-2.65	0.66	<.001	-3.95	-1.34	-1.33
Week 6 * Group	-3.18	0.66	<.001	-4.49	-1.87	-1.06
Week 8 * Group	-3.74	0.70	<.001	-5.13	-2.36	-0.94
**Daytime sleepiness** (*N*=192)						
Intercept	4.56	2.38	.06	-0.15	9.26	--
Gender=female	-0.70	0.71	.32	-2.11	0.70	-1.40
Racial minority	-0.03	0.91	.97	-1.83	1.76	-0.08
College education	-0.30	0.73	.68	-1.74	1.13	-0.61
Hourly worker	0.80	0.88	.36	-0.94	2.55	1.60
Frontline worker	9.09	4.40	.04	0.40	17.78	18.40
Mental health dx	1.97	0.81	.02	0.37	3.58	4.14
Sleep dx	1.70	0.81	.04	0.09	3.30	3.73
CCS enrolled	0.32	0.89	.72	-1.45	2.08	0.67
CCS attended	-1.15	1.29	.38	-3.70	1.41	-3.30
Group=Calm	-1.43	0.91	.12	-3.23	0.36	-2.86
Week 2	-0.01	0.36	.97	-0.71	0.69	-0.02
Week 4	-0.60	0.32	.06	-1.23	0.02	-1.50
Week 6	-0.92	0.32	<.001	-1.55	-0.30	-2.30
Week 8	-0.41	0.33	.21	-1.06	0.24	-1.02
Week 2 * Group	-0.47	0.51	.36	-1.49	0.54	-0.47
Week 4 * Group	-0.70	0.46	.12	-1.60	0.20	-0.35
Week 6 * Group	-0.91	0.46	.047	-1.81	-0.01	-0.30

Week 8 * Group	-1.73	0.47	<.001	-2.66	-0.79	-0.43

Note. SE = standard error; *p* = *p*-value; 95% CI LL and UL = 95% confidence interval lower limit and upper limit; *d* = Cohens *d*; CCS=Calm Coaching for Sleep. Complete cases were defined as participants who provided survey data at all five time points. Baseline (week 0) was the reference group for all time by group interaction terms.

Table S3.5. Estimates of group differences in changes in resilience over time (all available data; *N*=1026).

Parameter	Coefficient	*SE*	*p*	95% CI LL	95% CI UL	*d*
Intercept	3.97	0.19	<.001	3.59	4.35	--
Gender=female	-0.17	0.05	<.001	-0.27	-0.06	-0.34
Racial minority	-0.11	0.06	.07	-0.24	0.01	-0.25
College education	0.15	0.05	.01	0.04	0.25	0.30
Hourly worker	-0.32	0.06	<.001	-0.44	-0.20	-0.66
Frontline worker	0.14	0.24	.56	-0.34	0.62	0.30
Mental health dx	-0.60	0.06	<.001	-0.72	-0.49	-1.24
Sleep dx	0.00	0.06	.97	-0.12	0.13	0.00
CCS enrolled	-0.21	0.08	.01	-0.36	-0.06	-0.54
CCS attended	0.00	0.13	.99	-0.25	0.25	0.00
Group=Calm	-0.04	0.06	.45	-0.16	0.07	-0.08
Week 2	0.08	0.04	.03	0.01	0.16	0.20
Week 4	0.13	0.04	<.001	0.05	0.22	0.32
Week 6	0.13	0.04	<.001	0.05	0.21	0.32
Week 8	0.14	0.05	<.001	0.04	0.23	0.35
Week 2 * Group	0.01	0.05	.84	-0.09	0.11	0.01
Week 4 * Group	0.07	0.06	.24	-0.04	0.18	0.04
Week 6 * Group	0.05	0.06	.39	-0.06	0.16	0.02
Week 8 * Group	0.09	0.06	.16	-0.03	0.21	0.02

Note. SE = standard error; *p* = *p*-value; 95% CI LL and UL = 95% confidence interval lower limit and upper limit; *d* = Cohens *d*; CCS=Calm Coaching for Sleep. Resilience was measured using the Brief Resilience Scale. Baseline (week 0) was the reference group for all time by group interaction terms.

Table S3.6. Estimates of group differences in changes in resilience over time (complete cases; *N*=192).

Parameter	Coefficient	*SE*	*p*	95% CI LL	95% CI UL	*d*
Intercept	4.25	0.44	<.001	3.38	5.11	
Gender=female	0.00	0.13	.97	-0.25	0.26	0.00
Racial minority	-0.41	0.17	.02	-0.74	-0.08	-1.06
College education	0.30	0.13	.03	0.04	0.57	0.61
Hourly worker	-0.42	0.16	.01	-0.74	-0.10	-0.84
Frontline worker	-0.11	0.81	.89	-1.71	1.49	-0.22
Mental health dx	-0.47	0.15	<.001	-0.76	-0.17	-0.99
Sleep dx	-0.11	0.15	.45	-0.41	0.18	-0.24
CCS enrolled	-0.45	0.16	.01	-0.78	-0.13	-0.94
CCS attended	-0.26	0.24	.27	-0.73	0.21	-0.74
Group=Calm	-0.14	0.17	.40	-0.46	0.19	-0.28
Week 2	0.04	0.06	.49	-0.07	0.15	0.10
Week 4	0.06	0.06	.27	-0.05	0.18	0.15
Week 6	0.06	0.06	.27	-0.05	0.17	0.15
Week 8	0.10	0.06	.11	-0.02	0.21	0.25
Week 2 * Group	0.07	0.08	.37	-0.09	0.23	0.07
Week 4 * Group	0.20	0.08	.02	0.04	0.37	0.10
Week 6 * Group	0.15	0.08	.07	-0.01	0.31	0.05
Week 8 * Group	0.21	0.09	.02	0.04	0.38	0.05

Note. SE = standard error; *p* = *p*-value; 95% CI LL and UL = 95% confidence interval lower limit and upper limit; *d* = Cohens *d*; CCS=Calm Coaching for Sleep. Resilience was measured using the Brief Resilience Scale. Complete cases were defined as participants who provided survey data at all five time points. Baseline (week 0) was the reference group for all time by group interaction terms.

Table S3.7. Estimates of group differences in changes in work productivity and activity impairment over time (all available data).

Parameter	Coefficient	*SE*	*p*	95% CI LL	95% CI UL	*d*
**Absenteeism** (*N*=974)						
Intercept	10.19	2.98	<.001	4.35	16.04	
Gender=female	0.36	0.85	.67	-1.30	2.03	0.03
Racial minority	0.68	1.02	.51	-1.32	2.67	0.05
College education	-0.98	0.87	.26	-2.68	0.72	-0.07
Hourly worker	2.76	1.01	.01	0.77	4.76	0.20
Frontline worker	-6.62	4.15	.11	-14.77	1.53	-0.47
Mental health dx	4.42	0.95	<.001	2.55	6.29	0.31
Sleep dx	0.68	0.99	.49	-1.27	2.63	0.05
CCS enrolled	-2.24	1.19	.06	-4.58	0.09	-0.16
CCS attended	-2.48	1.84	.18	-6.10	1.13	-0.18
Group=Calm	-0.54	1.09	.62	-2.68	1.61	-0.04
Week 2	-0.18	1.24	.89	-2.62	2.27	-0.01
Week 4	-0.95	1.07	.38	-3.06	1.16	-0.07
Week 6	0.73	1.71	.67	-2.64	4.10	0.05
Week 8	-0.42	1.34	.75	-3.05	2.21	-0.03
Week 2 * Group	-0.13	1.71	.94	-3.48	3.22	-0.01
Week 4 * Group	-0.09	1.48	.95	-3.00	2.83	-0.01
Week 6 * Group	-0.96	2.42	.69	-5.73	3.80	-0.07
Week 8 * Group	-0.09	1.76	.96	-3.54	3.36	-0.01
**Presenteeism** (*N*=953)						
Intercept	23.67	5.53	<.001	12.82	34.53	
Gender=female	0.77	1.56	.62	-2.30	3.84	0.03
Racial minority	4.15	1.87	.03	0.48	7.83	0.16
College education	-2.44	1.60	.13	-5.58	0.70	-0.10
Hourly worker	6.24	1.85	<.001	2.60	9.88	0.24
Frontline worker	-7.43	7.26	.31	-21.67	6.81	-0.29
Mental health dx	16.67	1.76	<.001	13.22	20.12	0.65
Sleep dx	6.42	1.84	<.001	2.81	10.03	0.25
CCS enrolled	4.34	2.22	.051	-0.01	8.70	0.17
CCS attended	-4.91	3.49	.16	-11.76	1.94	-0.19
Group=Calm	-1.84	1.89	.33	-5.55	1.88	-0.07
Week 2	-4.00	1.84	.03	-7.61	-0.38	-0.16
Week 4	-3.09	1.78	.08	-6.59	0.40	-0.12
Week 6	-3.28	2.05	.11	-7.31	0.75	-0.13
Week 8	-3.19	2.13	.13	-7.38	1.00	-0.12
Week 2 * Group	3.84	2.53	.13	-1.13	8.82	0.15
Week 4 * Group	-2.54	2.47	.30	-7.39	2.31	-0.10
Week 6 * Group	-2.48	2.87	.39	-8.13	3.17	-0.10

Week 8 * Group	-2.54	2.80	.36	-8.05	2.96	-0.10
**Overall work impairment** (*N*=950)						
Intercept	27.14	5.95	<.001	15.47	38.82	
Gender=female	1.11	1.68	.51	-2.19	4.41	0.04
Racial minority	4.60	2.02	.02	0.63	8.56	0.17
College education	-3.06	1.72	.08	-6.44	0.32	-0.11
Hourly worker	7.72	2.01	<.001	3.78	11.67	0.28
Frontline worker	-8.10	8.04	.31	-23.88	7.67	-0.29
Mental health dx	18.32	1.89	<.001	14.61	22.03	0.66
Sleep dx	6.76	1.98	<.001	2.88	10.65	0.25
CCS enrolled	3.36	2.38	.16	-1.31	8.04	0.12
CCS attended	-6.05	3.74	.11	-13.40	1.30	-0.22
Group=Calm	-2.30	2.05	.26	-6.31	1.71	-0.08
Week 2	-4.67	1.98	.02	-8.56	-0.78	-0.17
Week 4	-3.66	1.96	.06	-7.52	0.20	-0.13
Week 6	-3.09	2.17	.15	-7.36	1.17	-0.11
Week 8	-3.12	2.27	.17	-7.59	1.34	-0.11
Week 2 * Group	4.25	2.72	.12	-1.09	9.60	0.15
Week 4 * Group	-2.17	2.72	.43	-7.51	3.18	-0.08
Week 6 * Group	-3.00	3.06	.33	-9.01	3.02	-0.11
Week 8 * Group	-2.37	2.99	.43	-8.23	3.50	-0.09
**Activity impairment** (*N*=1012)						
Intercept	24.23	5.88	<.001	12.68	35.78	
Gender=female	3.72	1.66	.03	0.46	6.97	0.13
Racial minority	4.69	1.97	.02	0.82	8.55	0.17
College education	-5.03	1.69	<.001	-8.35	-1.71	-0.18
Hourly worker	6.71	1.95	<.001	2.88	10.55	0.24
Frontline worker	-14.44	7.65	.06	-29.46	0.57	-0.51
Mental health dx	18.76	1.87	<.001	15.09	22.42	0.66
Sleep dx	8.58	1.95	<.001	4.75	12.42	0.30
CCS enrolled	8.02	2.35	<.001	3.39	12.64	0.28
CCS attended	-0.33	3.74	.93	-7.68	7.01	-0.01
Group=Calm	-1.08	1.95	.58	-4.91	2.74	-0.04
Week 2	-2.61	1.84	.16	-6.23	1.00	-0.09
Week 4	-4.74	1.70	.01	-8.09	-1.40	-0.17
Week 6	-2.47	2.07	.23	-6.53	1.59	-0.09
Week 8	-1.98	2.08	.34	-6.06	2.10	-0.07
Week 2 * Group	2.77	2.51	.27	-2.17	7.70	0.10
Week 4 * Group	-1.79	2.36	.45	-6.43	2.84	-0.06
Week 6 * Group	-4.11	2.89	.16	-9.79	1.57	-0.15
Week 8 * Group	-5.67	2.74	.04	-11.05	-0.30	-0.20

Note. SE = standard error; *p* = *p*-value; 95% CI LL and UL = 95% confidence interval lower limit and upper limit; *d* = Cohens *d*; CCS=Calm Coaching for Sleep. All outcomes were measured using the Work

Productivity and Activity Impairment Questionnaire. Baseline (week 0) was the reference group for all time by group interaction terms.

Table S3.8. Estimates of group differences in changes in work productivity and activity impairment over time (complete cases).

Parameter	Coefficient	*SE*	*p*	95% CI LL	95% CI UL	*d*
**Absenteeism** (*N*=188)						
Intercept	12.84	4.80	.01	3.35	22.34	
Gender=female	0.80	1.41	.57	-1.98	3.59	0.06
Racial minority	-0.91	1.79	.61	-4.45	2.62	-0.07
College education	-1.85	1.44	.20	-4.69	0.99	-0.15
Hourly worker	1.53	1.74	.38	-1.92	4.98	0.12
Frontline worker	-12.91	8.60	.14	-29.91	4.09	-1.05
Mental health dx	2.30	1.61	.15	-0.88	5.48	0.19
Sleep dx	-0.25	1.60	.88	-3.41	2.92	-0.02
CCS enrolled	-3.20	1.77	.07	-6.69	0.29	-0.26
CCS attended	-2.37	2.57	.36	-7.46	2.72	-0.19
Group=Calm	-0.78	2.15	.72	-5.02	3.46	-0.06
Week 2	0.52	1.62	.75	-2.67	3.71	0.04
Week 4	1.21	1.39	.38	-1.53	3.95	0.10
Week 6	3.20	2.32	.17	-1.39	7.79	0.26
Week 8	1.63	1.73	.35	-1.80	5.05	0.13
Week 2 * Group	-0.99	2.36	.67	-5.64	3.66	-0.08
Week 4 * Group	-2.35	2.03	.25	-6.35	1.64	-0.19
Week 6 * Group	-2.64	3.36	.43	-9.28	4.01	-0.21
Week 8 * Group	-2.40	2.49	.34	-7.31	2.52	-0.19
**Presenteeism** (*N*=180)						
Intercept	10.76	10.68	.32	-10.33	31.86	
Gender=female	0.72	3.11	.82	-5.43	6.87	0.03
Racial minority	11.01	3.96	.01	3.18	18.85	0.44
College education	-6.55	3.18	.04	-12.84	-0.26	-0.26
Hourly worker	6.93	3.86	.08	-0.71	14.56	0.28
Frontline worker	-3.04	19.10	.87	-40.80	34.72	-0.12
Mental health dx	20.15	3.56	<.001	13.11	27.19	0.80
Sleep dx	2.99	3.55	.40	-4.03	10.00	0.12
CCS enrolled	2.14	3.91	.59	-5.59	9.86	0.09
CCS attended	-3.83	5.68	.50	-15.05	7.39	-0.15
Group=Calm	3.27	4.79	.50	-6.17	12.71	0.13
Week 2	0.47	2.95	.87	-5.35	6.29	0.02
Week 4	2.87	2.68	.28	-2.41	8.16	0.11
Week 6	3.51	2.85	.22	-2.12	9.13	0.14
Week 8	0.69	3.03	.82	-5.30	6.67	0.03
Week 2 * Group	-4.93	4.29	.25	-13.40	3.53	-0.20
Week 4 * Group	-11.48	3.92	<.001	-19.21	-3.74	-0.46
Week 6 * Group	-10.54	4.12	.01	-18.66	-2.41	-0.42

Week 8 * Group	-12.32	4.36	.01	-20.93	-3.70	-0.49
**Overall work impairment** (*N*=181)						
Intercept	19.07	11.24	.09	-3.13	41.26	
Gender=female	1.05	3.28	.75	-5.43	7.53	0.04
Racial minority	9.85	4.17	.02	1.61	18.10	0.37
College education	-7.65	3.35	.02	-14.27	-1.02	-0.29
Hourly worker	7.47	4.07	.07	-0.58	15.52	0.28
Frontline worker	-10.73	20.11	.59	-50.49	29.03	-0.40
Mental health dx	20.89	3.75	<.001	13.48	28.31	0.78
Sleep dx	2.95	3.74	.43	-4.44	10.34	0.11
CCS enrolled	0.75	4.11	.85	-7.37	8.88	0.03
CCS attended	-5.44	5.98	.36	-17.27	6.38	-0.20
Group=Calm	2.88	5.03	.57	-7.05	12.80	0.11
Week 2	-0.05	3.08	.99	-6.12	6.02	0.00
Week 4	3.17	2.91	.28	-2.57	8.91	0.12
Week 6	4.48	3.02	.14	-1.47	10.43	0.17
Week 8	1.44	3.21	.65	-4.89	7.77	0.05
Week 2 * Group	-5.49	4.47	.22	-14.30	3.32	-0.21
Week 4 * Group	-12.52	4.24	<.001	-20.90	-4.15	-0.47
Week 6 * Group	-12.08	4.39	.01	-20.74	-3.43	-0.45
Week 8 * Group	-13.45	4.61	<.001	-22.55	-4.35	-0.51
**Activity impairment** (*N*=192)						
Intercept	16.63	11.62	.15	-6.31	39.58	
Gender=female	1.69	3.43	.62	-5.09	8.47	0.06
Racial minority	13.28	4.39	<.001	4.61	21.95	0.47
College education	-7.55	3.51	.03	-14.49	-0.62	-0.27
Hourly worker	6.44	4.26	.13	-1.98	14.87	0.23
Frontline worker	-10.19	21.24	.63	-52.16	31.78	-0.36
Mental health dx	24.33	3.92	<.001	16.57	32.08	0.86
Sleep dx	7.81	3.92	.048	0.06	15.56	0.28
CCS enrolled	3.23	4.31	.45	-5.29	11.75	0.11
CCS attended	-2.54	6.25	.69	-14.88	9.80	-0.09
Group=Calm	4.71	5.00	.35	-5.14	14.56	0.17
Week 2	1.54	2.94	.60	-4.26	7.33	0.05
Week 4	2.56	2.53	.31	-2.43	7.56	0.09
Week 6	4.23	2.78	.13	-1.24	9.70	0.15
Week 8	3.21	2.78	.25	-2.28	8.69	0.11
Week 2 * Group	-6.68	4.24	.12	-15.04	1.69	-0.24
Week 4 * Group	-12.70	3.66	<.001	-19.91	-5.49	-0.45
Week 6 * Group	-13.40	4.01	<.001	-21.29	-5.50	-0.47
Week 8 * Group	-17.14	4.02	<.001	-25.06	-9.21	-0.61

Note. SE = standard error; *p* = *p*-value; 95% CI LL and UL = 95% confidence interval lower limit and upper limit; *d* = Cohens *d*; CCS=Calm Coaching for Sleep. All outcomes were measured using the Work

Productivity and Activity Impairment Questionnaire. Complete cases were defined as participants who provided survey data at all five time points. Baseline (week 0) was the reference group for all time by group interaction terms.

Table S3.9. Estimates of group differences in changes in health care visits over time (all available data; *N*=1028).

Parameter	Coefficient	*SE*	*p*	95% CI LL	95% CI UL	*d*
Intercept	0.43	0.24	.07	-0.04	0.90	
Gender=female	0.14	0.07	.04	0.01	0.27	0.28
Racial minority	-0.04	0.08	.65	-0.19	0.12	-0.09
College education	0.11	0.07	.10	-0.02	0.25	0.22
Hourly worker	0.20	0.08	.01	0.05	0.36	0.41
Frontline worker	0.13	0.31	.67	-0.48	0.75	0.28
Mental health dx	0.38	0.08	<.001	0.23	0.53	0.79
Sleep dx	0.22	0.08	.01	0.06	0.37	0.50
CCS enrolled	0.06	0.10	.56	-0.13	0.24	0.16
CCS attended	-0.22	0.15	.14	-0.52	0.07	-0.98
Group=Calm	-0.11	0.08	.15	-0.26	0.04	-0.22
Week 4	-0.25	0.08	<.001	-0.41	-0.10	-0.62
Week 8	0.96	0.10	<.001	0.77	1.15	2.40
Week 4 * Group	0.15	0.11	.14	-0.05	0.36	0.08
Week 8 * Group	-0.93	0.13	<.001	-1.19	-0.68	-0.23

Note. SE = standard error; *p* = *p*-value; 95% CI LL and UL = 95% confidence interval lower limit and upper limit; *d* = Cohens *d*; CCS=Calm Coaching for Sleep. Health care visits determined by responses to the question "How many times have you seen a medical provider in the last four weeks?" Baseline (week 0) was the reference group for all time by group interaction terms.

Table S3.10. Estimates of group differences in changes in health care visits over time (complete cases; *N*=192).

Parameter	Coefficient	*SE*	*p*	95% CI LL	95% CI UL	*d*
Intercept	0.01	0.43	.99	-0.84	0.85	
Gender=female	0.11	0.13	.40	-0.14	0.35	0.22
Racial minority	-0.06	0.16	.70	-0.38	0.26	-0.16
College education	0.21	0.13	.11	-0.05	0.46	0.43
Hourly worker	0.24	0.16	.14	-0.07	0.55	0.48
Frontline worker	-0.08	0.78	.92	-1.63	1.46	-0.16
Mental health dx	0.50	0.14	<.001	0.22	0.79	1.05
Sleep dx	0.18	0.14	.21	-0.10	0.47	0.40
CCS enrolled	0.19	0.16	.22	-0.12	0.51	0.40
CCS attended	-0.14	0.23	.54	-0.60	0.31	-0.40
Group=Calm	0.00	0.19	.99	-0.38	0.37	0.00
Week 4	-0.14	0.12	.25	-0.38	0.10	-0.35
Week 8	1.05	0.14	<.001	0.77	1.33	2.62
Week 4 * Group	0.02	0.18	.93	-0.34	0.37	0.01
Week 8 * Group	-1.05	0.21	<.001	-1.46	-0.65	-0.26

Note. SE = standard error; *p* = *p*-value; 95% CI LL and UL = 95% confidence interval lower limit and upper limit; *d* = Cohens *d*; CCS=Calm Coaching for Sleep. Health care visits determined by responses to the question "How many times have you seen a medical provider in the last four weeks?" Complete cases were defined as participants who provided survey data at all five time points. Baseline (week 0) was the reference group for all time by group interaction terms.

Table S3.11. Estimates of group differences in changes in work impairment costs over time (all available data; *N*=770)

Parameter	Coefficient	*SE*	*p*	95% CI LL	95% CI UL	*d*
Intercept	386.56	34.93	<.001	317.99	455.14	--
Gender=female	14.81	21.68	0.49	-27.75	57.37	0.04
Racial minority	50.13	24.66	0.04	1.71	98.55	0.14
College education	-20.35	21.92	0.35	-63.38	22.68	-0.06
Hourly worker	-161.93	25.16	<.001	-211.31	-112.55	-0.46
Frontline worker	-68.09	25.72	0.01	-118.59	-17.60	-0.19
Mental health dx	142.65	24.31	<.001	94.94	190.37	0.40
Sleep dx	63.42	25.42	0.01	13.51	113.32	0.18
CCS enrolled	75.95	30.80	0.01	15.48	136.41	0.21
CCS attended	71.78	49.09	0.14	-24.63	168.19	0.20
Group=Calm	-23.37	24.82	0.35	-72.09	25.35	-0.07
Week 2	-50.91	25.31	0.04	-100.65	-1.18	-0.14
Week 4	-32.57	27.41	0.24	-86.45	21.32	-0.09
Week 6	-37.46	30.77	0.22	-98.01	23.09	-0.11
Week 8	-24.70	31.16	0.43	-85.99	36.58	-0.07
Week 2 * Group	52.43	34.85	0.13	-16.05	120.92	0.15
Week 4 * Group	-33.30	38.21	0.38	-108.43	41.82	-0.09
Week 6 * Group	-24.05	43.61	0.58	-109.87	61.76	-0.07
Week 8 * Group	-28.41	40.80	0.49	-108.64	51.82	-0.08

Note. SE=standard error; *p*=*p*-value; 95% CI LL and UL=95% confidence interval lower limit and upper limit; *d*=Cohens *d*; CCS=Calm Coaching for Sleep. Work impairment costs were calculated by multiplying an employee's weekly pay by their overall work impairment percentage (i.e., absenteeism and presenteeism). Baseline (week 0) was the reference group for all time by group interaction terms.

Table S3.12. Estimates of group differences in changes in work impairment costs over time (complete cases; *N*=150)

Parameter	Coefficient	*SE*	*p*	95% CI LL	95% CI UL	*d*
Intercept	304.81	67.23	<.001	172.17	437.45	--
Gender=female	56.96	43.47	0.19	-28.95	142.88	0.17
Racial minority	85.50	54.84	0.12	-22.91	193.90	0.26
College education	-39.70	43.51	0.36	-125.71	46.31	-0.12
Hourly worker	-126.76	52.53	0.02	-230.58	-22.93	-0.38
Frontline worker	-61.81	50.46	0.22	-161.55	37.93	-0.19
Mental health dx	185.41	49.79	<.001	86.99	283.84	0.56
Sleep dx	-22.56	49.07	0.65	-119.56	74.45	-0.07
CCS enrolled	30.17	54.22	0.58	-77.00	137.34	0.09
CCS attended	99.65	78.97	0.21	-56.42	255.73	0.30
Group=Calm	53.86	60.68	0.38	-65.77	173.49	0.16
Week 2	-5.84	35.39	0.87	-75.69	64.01	-0.02
Week 4	46.97	38.16	0.22	-28.27	122.20	0.14
Week 6	19.62	37.52	0.60	-54.36	93.60	0.06
Week 8	16.47	40.20	0.68	-62.87	95.80	0.05
Week 2 * Group	-49.67	51.33	0.33	-150.98	51.65	-0.15
Week 4 * Group	-141.83	55.87	0.01	-251.97	-31.70	-0.43
Week 6 * Group	-97.49	54.62	0.08	-205.20	10.21	-0.29
Week 8 * Group	-155.66	57.76	0.01	-269.66	-41.66	-0.47

Note. SE=standard error; *p*=*p*-value; 95% CI LL and UL=95% confidence interval lower limit and upper limit; *d*=Cohens *d*; CCS=Calm Coaching for Sleep. Work impairment costs were calculated by multiplying an employee's weekly pay by their overall work impairment percentage (i.e., absenteeism and presenteeism). Baseline (week 0) was the reference group for all time by group interaction terms.
