# Supplementary material for: Testing the Pragmatic Effectiveness of a Consumer-Based Mindfulness Mobile App in the Workplace: Randomized Controlled Trial
Source: JMIR Mhealth Uhealth. 2022 Sep 28;10(9):e38903. doi: 10.2196/38903 (PMC9557765; doi:10.2196/38903)
Supplement: Multimedia Appendix 4 [file mhealth_v10i9e38903_app4.pdf]

Figure S4.1. Average number of Calm sessions per week completed by employees during the intervention

	Sessions per week							
	Week 1		Week 2		Week 3		Week 4	
	M	SD	M	SD	M	SD	M	SD
Meditation	2.55	4.71	2.76	4.80	2.67	5.10	2.38	5.13
Sleep Stories	0.31	1.23	0.33	1.29	0.46	1.83	0.33	1.79
Music	2.00	23.09	0.57	3.28	1.34	8.45	1.54	10.81
Soundscapes	0.23	1.77	1.23	8.87	1.26	11.72	1.74	13.68
Calm Body	0.03	0.25	0.00	0.00	0.06	0.58	0.09	0.65
Masterclass	0.39	2.54	0.19	1.38	0.29	1.65	0.19	1.38
Spark	0.03	0.23	0.02	0.13	0.01	0.09	0.01	0.11
Movement	0.00	0.00	0.00	0.00	0.01	0.09	0.00	0.00
Any Calm session	5.52	24.65	5.10	11.79	6.10	16.46	6.28	19.13

Figure S4.1, continued. Average number of Calm sessions per week completed by employees during the intervention

	Sessions per week							
	Week 5		Week 6		Week 7		Week 8	
	M	SD	M	SD	M	SD	M	SD
Meditation	1.54	4.28	1.68	4.37	1.27	4.19	1.00	3.63
Sleep Stories	0.39	2.11	0.43	2.19	0.30	1.99	0.40	2.14
Music	3.40	23.61	2.73	21.51	1.20	9.21	0.90	5.44
Soundscapes	2.32	20.49	1.92	19.77	1.94	19.23	2.12	20.02
Calm Body	0.02	0.13	0.04	0.53	0.03	0.34	0.02	0.34
Masterclass	0.10	0.79	0.10	0.64	0.01	0.20	0.01	0.21
Spark	0.01	0.11	0.01	0.09	0.00	0.00	0.00	0.00
Movement	0.00	0.00	0.00	0.00	0.02	0.21	0.02	0.23
Any Calm session	7.77	32.22	6.91	30.35	4.76	22.13	4.48	21.51

Figure S4.2. Average minutes of Calm usage per week by employees during the intervention

	Minutes per week							
	Week 1		Week 2		Week 3		Week 4	
	M	SD	M	SD	M	SD	M	SD
Meditation	29.34	57.16	33.86	62.37	33.64	66.53	31.94	74.25
Sleep Stories	10.72	42.28	11.24	43.34	15.78	62.68	11.58	63.74
Music	9.09	73.04	7.99	55.68	16.53	98.55	14.15	102.91
Soundscapes	6.76	53.05	36.83	265.64	37.56	351.14	51.97	409.21
Calm Body	0.21	2.03	0.00	0.00	0.58	5.15	0.72	5.07
Masterclass	5.08	37.54	1.58	9.81	3.83	21.91	2.41	18.09
Spark	0.36	3.60	0.19	1.48	0.15	1.69	0.22	1.98
Movement	0.00	0.00	0.00	0.00	0.05	0.53	0.00	0.00
Any Calm use	61.55	147.35	91.70	296.48	108.10	387.55	112.98	444.39

Figure S4.2., continued. Average minutes of Calm usage per week by employees during the intervention

	Minutes per week							
	Week 5		Week 6		Week 7		Week 8	
	M	SD	M	SD	M	SD	M	SD
Meditation	23.59	73.58	25.19	73.17	16.00	57.28	11.81	42.17
Sleep Stories	14.08	78.31	15.64	80.40	10.32	68.54	13.93	73.61
Music	18.29	116.22	19.92	136.61	12.88	81.17	14.87	91.74
Soundscapes	69.40	613.80	57.34	592.28	57.95	575.98	63.55	599.67
Calm Body	0.17	1.31	0.27	3.14	0.16	1.97	0.13	1.97
Masterclass	1.17	9.59	0.99	6.73	0.12	1.84	0.13	1.91
Spark	0.17	1.49	0.11	1.24	0.00	0.00	0.00	0.00
Movement	0.00	0.00	0.00	0.00	0.10	1.18	0.14	1.28
Any Calm use	126.88	641.38	119.46	623.66	97.53	595.06	104.56	616.93
